# Global, regional, and national burden of Alzheimer's disease and other dementias, 1990–2016: a systematic analysis for the Global Burden of Disease Study 2016

**DOI:** 10.1016/S1474-4422(18)30403-4

**Published:** 2019-01

**Authors:** GBD 2016 Dementia Collaborators, Emma Nichols, Cassandra E I Szoeke, Stein Emil Vollset, Nooshin Abbasi, Foad Abd-Allah, Jemal Abdela, Miloud Taki Eddine Aichour, Rufus O Akinyemi, Fares Alahdab, Solomon W Asgedom, Ashish Awasthi, Suzanne L Barker-Collo, Bernhard T Baune, Yannick Béjot, Abate B Belachew, Derrick A. Bennett, Belete Biadgo, Ali Bijani, Muhammad Shahdaat Bin Sayeed, Carol Brayne, David O Carpenter, Félix Carvalho, Ferrán Catalá-López, Ester Cerin, Jee-Young J Choi, Anh Kim Dang, Meaza G Degefa, Shirin Djalalinia, Manisha Dubey, Eyasu Ejeta Duken, David Edvardsson, Matthias Endres, Sharareh Eskandarieh, Andre Faro, Farshad Farzadfar, Seyed-Mohammad Fereshtehnejad, Eduarda Fernandes, Irina Filip, Florian Fischer, Abadi K Gebre, Demeke Geremew, Maryam Ghasemi-Kasman, Elena V. Gnedovskaya, Rajeev Gupta, Vladimir Hachinski, Tekleberhan B Hagos, Samer Hamidi, Graeme J. Hankey, Josep M Haro, Simon I Hay, Seyed Sina N Irvani, Ravi P Jha, Jost B. Jonas, Rizwan Kalani, André Karch, Amir Kasaeian, Yousef Saleh Khader, Ibrahim A Khalil, Ejaz Ahmad Khan, Tripti Khanna, Tawfik A M Khoja, Jagdish Khubchandani, Adnan Kisa, Katarzyna Kissimova-Skarbek, Mika Kivimäki, Ai Koyanagi, Kristopher J Krohn, Giancarlo Logroscino, Stefan Lorkowski, Marek Majdan, Reza Malekzadeh, Winfried März, João Massano, Getnet Mengistu, Atte Meretoja, Moslem Mohammadi, Maryam Mohammadi-Khanaposhtani, Ali H Mokdad, Stefania Mondello, Ghobad Moradi, Gabriele Nagel, Mohsen Naghavi, Gurudatta Naik, Long H Nguyen, Trang H Nguyen, Yirga L Nirayo, Molly R Nixon, Richard Ofori-Asenso, Felix A Ogbo, Andrew T Olagunju, Mayowa O Owolabi, Songhomitra Panda-Jonas, Valéria M de Azeredo Passos, David M. Pereira, Gabriel D Pinilla-Monsalve, Michael A Piradov, Constance D Pond, Hossein Poustchi, Mostafa Qorbani, Amir Radfar, Robert C Reiner, Stephen R Robinson, Gholamreza Roshandel, Ali Rostami, Tom C Russ, Perminder S Sachdev, Hosein Safari, Saeid Safiri, Ramesh Sahathevan, Yahya Salimi, Maheswar Satpathy, Monika Sawhney, Mete Saylan, Sadaf G. Sepanlou, Azadeh Shafieesabet, Masood A Shaikh, Mohammad Ali Sahraian, Mika Shigematsu, Rahman Shiri, Ivy Shiue, João P Silva, Mari Smith, Soheila Sobhani, Dan J Stein, Rafael Tabarés-Seisdedos, Marcos R Tovani-Palone, Bach X Tran, Tung Thanh Tran, Amanuel T Tsegay, Irfan Ullah, Narayanaswamy Venketasubramanian, Vasily Vlassov, Yuan-Pang Wang, Jordan Weiss, Ronny Westerman, Tissa Wijeratne, Grant M.A. Wyper, Yuichiro Yano, Ebrahim M Yimer, Naohiro Yonemoto, Mahmoud Yousefifard, Zoubida Zaidi, Zohreh Zare, Theo Vos, Valery L. Feigin, Christopher J L Murray

## Abstract

**Background:**

The number of individuals living with dementia is increasing, negatively affecting families, communities, and health-care systems around the world. A successful response to these challenges requires an accurate understanding of the dementia disease burden. We aimed to present the first detailed analysis of the global prevalence, mortality, and overall burden of dementia as captured by the Global Burden of Diseases, Injuries, and Risk Factors (GBD) Study 2016, and highlight the most important messages for clinicians and neurologists.

**Methods:**

GBD 2016 obtained data on dementia from vital registration systems, published scientific literature and surveys, and data from health-service encounters on deaths, excess mortality, prevalence, and incidence from 195 countries and territories from 1990 to 2016, through systematic review and additional data-seeking efforts. To correct for differences in cause of death coding across time and locations, we modelled mortality due to dementia using prevalence data and estimates of excess mortality derived from countries that were most likely to code deaths to dementia relative to prevalence. Data were analysed by standardised methods to estimate deaths, prevalence, years of life lost (YLLs), years of life lived with disability (YLDs), and disability-adjusted life-years (DALYs; computed as the sum of YLLs and YLDs), and the fractions of these metrics that were attributable to four risk factors that met GBD criteria for assessment (high body-mass index [BMI], high fasting plasma glucose, smoking, and a diet high in sugar-sweetened beverages).

**Findings:**

In 2016, the global number of individuals who lived with dementia was 43·8 million (95% uncertainty interval [UI] 37·8–51·0), increased from 20.2 million (17·4–23·5) in 1990. This increase of 117% (95% UI 114–121) contrasted with a minor increase in age-standardised prevalence of 1·7% (1·0–2·4), from 701 cases (95% UI 602–815) per 100 000 population in 1990 to 712 cases (614–828) per 100 000 population in 2016. More women than men had dementia in 2016 (27·0 million, 95% UI 23·3–31·4, *vs* 16.8 million, 14.4–19.6), and dementia was the fifth leading cause of death globally, accounting for 2·4 million (95% UI 2·1–2·8) deaths. Overall, 28·8 million (95% UI 24·5–34·0) DALYs were attributed to dementia; 6·4 million (95% UI 3·4–10·5) of these could be attributed to the modifiable GBD risk factors of high BMI, high fasting plasma glucose, smoking, and a high intake of sugar-sweetened beverages.

**Interpretation:**

The global number of people living with dementia more than doubled from 1990 to 2016, mainly due to increases in population ageing and growth. Although differences in coding for causes of death and the heterogeneity in case-ascertainment methods constitute major challenges to the estimation of the burden of dementia, future analyses should improve on the methods for the correction of these biases. Until breakthroughs are made in prevention or curative treatment, dementia will constitute an increasing challenge to health-care systems worldwide.

**Funding:**

Bill & Melinda Gates Foundation.

## Introduction

Alzheimer's disease and other dementias are a major and increasing global health challenge, with 40–50 million people currently living with dementia.^1^, ^2^, ^3^ Care and support of patients with dementia has wide-ranging consequences for families, health-care systems, and society as a whole.^4^ There is growing evidence of risk factors for dementia, which shows that lifestyle and other interventions might, if implemented effectively, contribute to delaying the onset and reducing the future number of people who have dementia.^5^, ^6^, ^7^, ^8^, ^9^ Changes in risk factor exposures might account for several cohort studies documenting a reduction in age-specific incidence rates in their study populations.^3^, ^6^, ^10^, ^11^

The Global Burden of Diseases, Injuries, and Risk Factors (GBD) Study uses a systematic method to analyse fatal and non-fatal health losses to facilitate comparisons across countries and diseases. Annual updates of results quantify mortality, prevalence, incidence, and non-fatal health losses for more than 300 diseases and injuries by age and sex from 1990 for 195 countries and territories and many subnational locations, such as Mexican states or provinces of China. Although other efforts to estimate dementia prevalence^1^, ^2^, ^12^ have used meta-analytic strategies to synthesise the available data, these analyses did not attempt to reconcile and make the combined best use of different types of data on incidence, prevalence, excess mortality, and causes of death reported in literature sources, claims databases, and vital registration systems. Furthermore, GBD assesses the strength of causal evidence for risks and includes estimates of attributable disease burden from risk factors using a comparative risk assessment framework.

**Research in context**

**Evidence before this study**

Over the past decade, there have been substantial research efforts to describe the global epidemiology of Alzheimer's disease and other dementias. Other efforts to summarise the evidence, including the World Dementia Report, have compiled evidence through systematic reviews and meta-analyses, but they report only on dementia prevalence by world region. The Global Burden of Diseases, Injuries, and Risk Factors (GBD) Study has published regular updates of estimates for dementia in 195 countries and territories and subnational locations, such as the provinces of China and states in countries such as Brazil, the USA, and Mexico. However, there has been no dedicated and detailed publication of GBD methods and estimates for dementia. GBD 2016 estimates for dementia incorporated 43 new data sources on the prevalence and incidence of dementia, updating a previous review that covered 1980 to 2015; new sources were identified through a systematic review of English-language articles published in PubMed from 23 Jan, 2015, to Oct 7, 2016, with the search terms “dementia”[Title/Abstract] AND (“prevalence” OR “incidence”)[Title/Abstract].

**Added value of this study**

GBD 2016 added 5-year age groups from ages 80 to 95 years to replace the oldest category (≥80 years) used in GBD 2015, providing more detailed estimates where the burden from dementia was highest. Our report examined more closely the methods, results, and limitations specific to Alzheimer's disease and other dementias with the aim of making this information more accessible to clinicians and researchers. The data informing estimates were heterogeneous, with 230 different diagnostic procedures across 237 studies. Over time, the global age-standardised prevalence was fairly stable: 701 cases (95% uncertainty interval [UI] 602–815) per 100 000 population in 1990 and 712 cases (614–828) per 100 000 population in 2016, constituting an increase of 1·7% (95% UI 1·0–2·4). However, because of population ageing and growth, the number of people affected by dementia more than doubled since 1990 and almost 44 million prevalent cases were estimated globally in 2016. Age-standardised prevalence was 1·17 (1·17–1·18) times higher in females than in males. We also estimated that 22·3% (11·8–35·1) of the total global DALYs due to dementia in 2016 could be attributed to the four modifiable risk factors that met GBD criteria for assessment (high body-mass index, high fasting plasma glucose, a diet high in sugar-sweetened beverages, and smoking).

**Implications of all the available evidence**

This analysis identified substantial heterogeneity in case-ascertainment methods throughout the dementia literature, highlighting the need for more consistency in future research. Nevertheless, there is no doubt about the striking increase in the numbers of individuals living with dementia since 1990 due to ageing and population growth. Despite some evidence indicating small decreases in age-specific incidence, without a major scientific breakthrough the continuation of sweeping demographic trends in population ageing and growth will lead to further increases in the number of people living with dementia. With limited scope for prevention and the absence of an effective disease-modifying treatment, the burden on caregivers and the parts of health-care systems devoted to care of the elderly will continue to increase rapidly.

Global data and cross-country comparisons might help further current understanding of complex and multifactorial diseases such as dementia. The capacity of GBD to examine patterns across countries creates a unique opportunity to identify populations with different trends that in turn could reveal clustered risk or environmental factors, providing empirical evidence about factors that affect neurodegenerative diseases.^4^ Such findings could provide insights that can supplement and aid ongoing research, and also allow estimates of the future effect of dementia expected from rapidly ageing populations across the world. To facilitate further exploration of these patterns, we present an analysis of GBD 2016 data with the aim of articulating the key methods, results, and limitations pertaining to dementia estimation.

## Methods

### Overview and data sources

General methods of GBD, including methods for calculating the Socio-demographic Index (SDI), a composite indicator combining income per person, education, and fertility, can be found in the general methods overview (appendix) and in the GBD 2016 overview papers.^13^, ^14^, ^15^, ^16^ In this Article, we have highlighted the methods pertaining to processes specific to the estimation of Alzhemier's disease and other dementias (hereafter referred to as dementia).

For the GBD analyses, the reference case definitions were either those from the Diagnostic and Statistical Manual of Mental Disorders (DSM; DSM-III, DSM-IV, or DSM-5), which are used in surveys and cohort studies, or those from the International Classification of Diseases (ICD; ICD-8, ICD-9, and ICD-10), which are used in vital registration and claims data sources.^17^, ^18^ For GBD analyses of dementia, the relevant ICD-9 codes were 290, 291.2, 291.8, 294, and 331, and the relevant ICD-10 codes were F00, F01, F02, F03, G30, and G31. In GBD, for each disease a reference case definition was chosen that represents the most recent consensus or the most commonly used definition. Data based on alternative case definitions were adjusted if systematic bias was detected. We included 237 sources of data from the scientific literature, and 3 years of medical claims data from the USA. We identified 176 sources reporting on prevalence, covering 17 of 21 world regions, and 64 sources on incidence, covering ten of 21 world regions (appendix). No input data were available for Oceania, central Asia, eastern Europe, or southern sub-Saharan Africa.

We could not adjust the data for different study designs or case definitions because of the extreme heterogeneity in case-ascertainment methods. We identified 230 different methods of screening and diagnosis in the 237 scientific literature sources. Data points that had an age range of greater than 20 years were split into new data points for the 5-year age groups within the age range, using the age pattern from the USA as the country for which we had the most detailed information.

### Natural-history model

We did not use the typical GBD cause of death ensemble modelling (CODEm) approach for dementia because we noted large inconsistencies between cause of death data and prevalence data over time and between countries. Data from the US Vital Registration system showed that the age-standardised rates of deaths from dementia between 1990 and 2016 increased by a factor of five, whereas there has been no corresponding increase in the prevalence of dementia over the same period.^19^, ^20^, ^21^ Additionally, the highest age-standardised death rates were more than 50 times higher than the lowest age-standardised death rates across different locations for 2016, possibly indicating that the practice of coding a death to dementia as an underlying cause of death has not been consistent over time or between countries. To adjust for this bias, we jointly modelled cause of death and non-fatal outcomes for dementia. We first ran an initial cause of death model using CODEm, and an initial non-fatal model using DisMod-MR 2.1, the Bayesian meta-regression tool used in most non-fatal models for GBD.^22^, ^23^ DisMod-MR 2.1 enforces consistency between the different parameters, because incidence determines the inflow into the pool of prevalent cases and excess mortality determines outflow via death. The rates of inflow and outflow then determine the average duration of disease. Both DisMod MR 2.1 and CODEm use covariates and borrow strength from locations in the same region that have data to make estimates for locations where there are no input data.

The initial CODEm model included 16 226 site-years of data (ie, data for a unique combination of location and calendar year). The covariates used in the initial CODEm model included diabetes prevalence, mean cholesterol, and mean body-mass index (BMI; full list in the appendix). The initial DisMod-MR 2.1 model included settings of no remission (ie, no cure), and no incidence before age 40 years. We also excluded incidence data from the model, because data for incidence and prevalence were inconsistent. In many locations, the incidence data combined with mortality estimates suggested a prevalence higher than the available prevalence data, and the model fitted above the prevalence data and below the incidence data. Because measurement of prevalent cases of dementia probably has less error than measurement of incident cases, we decided to exclude incidence data and rely on prevalence data.

We let DisMod-MR 2.1 adjust medical claims data to correct for under-reporting compared with survey data. We used average years of education in the population aged 15 years and older and smoking prevalence as predictive covariates, because these risk factors for dementia have a large evidence base.^24^, ^25^ From the initial model results we identified the locations with high-quality vital registration systems that most often coded to dementia as a cause of death per prevalent case in the most recent year of estimates. For GBD 2016, these locations were the USA, Finland, Sweden, and Puerto Rico. We then used the log-transformed ratio between cause-specific mortality and prevalence for these locations to run a fixed-effects linear regression with dummy variables on 5-year age bins and sex. Because the ratio of cause-specific mortality and prevalence is an estimate of excess mortality rate, we used the results of this regression to predict data inputs for every GBD location and all years in a second DisMod-MR 2.1 model. As an exception, we retained the 2016 ratios of cause-specific mortality and prevalence for the four locations used in the regression analysis but assumed the 2016 ratios applied to the whole 1990–2016 period. Otherwise, the model included the same assumptions and covariates as the initial DisMod-MR 2.1 model. We used the prevalence outputs from this model to estimate years lived with disability (YLDs). We also used the cause-specific mortality estimated from this model for our final cause-specific mortality results.

### YLDs and risk estimation

To derive YLDs for dementia, we first divided dementia prevalence into three severity categories. We used data from a systematic review of studies reporting on the Clinical Dementia Rating Scale (CDR)^26^ and pooled proportions of mild (CDR 1), moderate (CDR 2), and severe dementia (CDR 3) in random-effect meta-analyses. We did this separately for ages 40–69, 70–79, and 80 years or more. For GBD 2016, we included seven studies identified through systematic review published from 1980 to 2015, covering two world regions. To calculate YLDs, we multiplied the prevalence at each severity level by the corresponding disability weight^24^ and further corrected for comorbidity using a simulation assigning all non-fatal outcomes to hypothetical individuals for each age group, sex, location, and year.^27^

We made estimates for high BMI, high fasting plasma glucose, smoking (including all smoked tobacco products), and diet high in sugar-sweetened beverages as risk factors for dementia. For each of these risk factors we set a theoretical minimum exposure level (TMREL) at which the risk of health outcomes is lowest. The TMREL was set to greater than 20 and less than 25 kg/m^2^ for BMI; greater than 4·5 and less than 5·4 mmol/L for high fasting plasma glucose; zero for smoking; and less than 5g/day for sugar-sweetened beverages.

Criteria for inclusion as a risk factor in GBD included sufficient evidence of a causal relationship, availability of exposure data, and potential for modification.^14^ Although physical inactivity and absence of education were not assessed for inclusion as risk factors for dementia in GBD 2016, these will be considered in upcoming rounds. We calculated population-attributable fractions based on relative risk data, exposure data, and a theoretical minimum level of exposure. When aggregating risks, we assumed a multiplicative function and accounted for instances where one risk was mediated through another. Additional details on risk factor calculations are in the GBD 2016 risk factor overview paper.^14^

### Compilation of results

Years of life lost (YLLs) were calculated by multiplying the reference life expectancy at each age, taken from the GBD reference life table, by the number of deaths in each age group.^28^ Disability-adjusted life years (DALYs) were then calculated as the sum of YLLs and YLDs. Age-standardised rates were calculated using the GBD world population standard.^29^ Uncertainty was propagated through all calculations by sampling 1000 draws at each step of the calculations, which enabled us to carry through uncertainty from multiple sources, including input data, corrections of measurement error, and estimates of residual non-sampling error. 95% uncertainty intervals (95% UI) were defined as the 25th and 975th values of the ordered draws. Any report on significant differences was based on the 95% UI of the difference not including zero.

### Role of the funding source

The funder of the study had no role in study design, data collection, data analysis, data interpretation, or writing of the report. All authors had full access to all the data in the study and had final responsibility for the decision to submit for publication.

## Results

**Figure 1:**
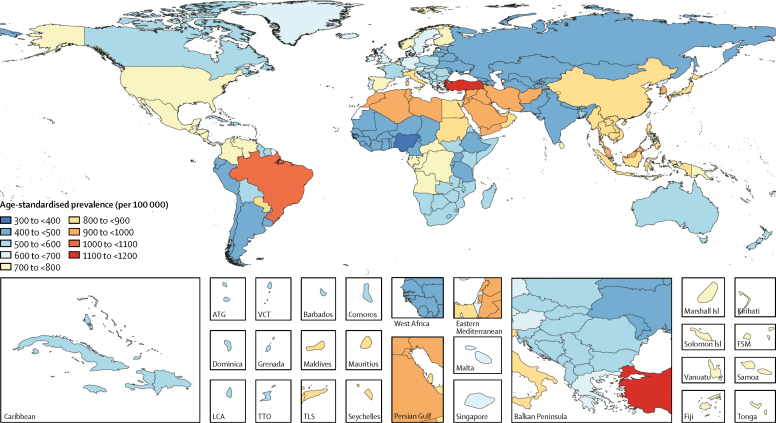
Age-standardised prevalence for Alzheimer's disease and other dementias per 100 000 population by location for both sexes, 2016 ATG=Antigua and Barbuda. FSM=Federated States of Micronesia. Isl=Islands. LCA=Saint Lucia. TLS=Timor-Leste. TTO=Trinidad and Tobago. VCT=Saint Vincent and the Grenadines.

Between 1990 and 2016, the number of prevalent dementia cases increased by 117% (95% UI 114–121), from 20·2 million (17·4–23·5) in 1990 to 43·8 million (37·8–51·0) in 2016, whereas there was an increase of only 1·7% (95% UI 1·0–2·4) in age-standardised prevalence, from 701 (602–815) per 100 000 population in 1990 to 712 (614–828) per 100 000 population in 2016. For all-age prevalence over the same period, there was an increase of 54·7% (95% UI 52·1–57·5), from 383 (330–447) per 100 000 population in 1990 to 593 (511–690) per 100 000 population in 2016. The percentage change from 1990 to 2016 in age-standardised prevalence was highest for the high-middle SDI quintile (table). Age-standardised prevalence varied by a factor of three across countries in 2016. Turkey had the highest age-standardised prevalence (1192 [95% UI 1007–1405] cases per 100 000 population), followed by Brazil (1037, 882–1220). Nigeria (397, 335–462) and Ghana (406, 342–483) had the lowest age-standardised prevalence estimates (figure 1).

**Table:** Deaths, prevalence, and DALYs for Alzheimer's disease and other dementias in 2016, and percentage change of age-standardised rates by location, 1990–2016

	**Deaths (95% UI)**		**Prevalence (95% UI)**		**DALYs (95% UI)**	
	2016 counts	Percentage change in age-standardised rates, 1990–2016	2016 counts	Percentage change in age-standardised rates, 1990–2016	2016 counts	Percentage change in age-standardised rates, 1990–2016
**Global**	**2 382 129 (2 060 410 to 2 777 610)**	**3·6 (1·1 to 5·6)**	**43 835 665 (37 756 336 to 51 028 051)**	**1·7 (1·0 to 2·4)**	**28 764 110 (24 510 789 to 33 952 387)**	**2·1 (0·1 to 3·8)**
High SDI	969 191 (844 580 to 1 104 900)	2·5 (0·2 to 5·2)	15 164 211 (13 282 384 to 17 306 959)	0·3 (−1·2 to 1·7)	9 886 396 (8 517 721 to 11 471 661)	−0·3 (−2·3 to 2·0)
High-middle SDI	448 457 (383 293 to 526 151)	4·1 (−0·6 to 9·1)	8 858 166 (7 587 609 to 10 305 640)	8·1 (6·7 to 10·4)	5 561 354 (4 692 609 to 6 539 404)	4·4 (0·4 to 8·6)
Middle SDI	658 727 (561 952 to 780 604)	0·9 (−4·5 to 5·0)	13 200 041 (11 201 343 to 15 593 470)	1·5 (0·7 to 2·3)	8 905 818 (7 506 192 to 10 630 061)	0·8 (−3·4 to 3·7)
Low-middle SDI	246 768 (206 143 to 300 717)	9·6 (1·7 to 16·5)	5 348 335 (4 509 649 to 6 326 468)	−2·7 (−3·4 to −2·1)	3 530 640 (2 942 604 to 4 280 829)	5·6 (0·3 to 10·6)
Low SDI	57 730 (48 159 to 71 752)	8·8 (2·8 to 15·8)	1 110 454 (934 598 to 1 315 691)	−3·0 (−3·9 to −1·9)	844 222 (696 157 to 1 031 439)	6·0 (1·5 to 11·0)
**High-income North America**	**260 080 (233 175 to 288 591)**	**9·9 (3·3 to 16·6)**	**4 347 849 (3 975 725 to 4 734 147)**	**−1·6 (−7·3 to 4·9)**	**2 688 057 (2 397 360 to 3 011 096)**	**2·5 (−3·4 to 8·9)**
Canada	21 180 (18 219 to 24 243)	−1·4 (−9·2 to 6·9)	317 027 (280 886 to 352 468)	−5·9 (−10·1 to −1·0)	214 389 (184 640 to 244 637)	−4·6 (−11·5 to 3·3)
Greenland	5 (4 to 7)	−2·0 (−16·1 to 13·4)	151 (125 to 178)	−4·0 (−7·1 to −0·5)	99 (76 to 127)	−4·1 (−16·1 to 9·1)
USA	238 895 (214 341 to 264 774)	11·6 (4·4 to 19·1)	4 029 450 (3 696 312 to 4 387 981)	−0·5 (−6·4 to 6·4)	2 473 390 (2 204 607 to 2 768 700)	3·8 (−2·5 to 10·8)
**Australasia**	**17 668 (15 110 to 20 461)**	**−6·2 (−13·0 to 1·7)**	**251 413 (217 349 to 287 182)**	**−9·1 (−15·4 to −4·0)**	**176 255 (151 230 to 206 647)**	**−8·2 (−14·5 to −1·2)**
Australia	14 977 (12 862 to 17 417)	−6·0 (−14·2 to 3·7)	211 208 (183 061 to 240 574)	−9·2 (−16·5 to −3·1)	148 229 (127 148 to 173 753)	−8·3 (−15·6 to 0·2)
New Zealand	2691 (2226 to 3250)	−7·0 (−15·6 to 1·9)	40 206 (33 855 to 47 870)	−8·4 (−15·1 to −3·5)	28 026 (23 231 to 34 113)	−7·6 (−14·9 to 0·1)
**High-income Asia-Pacific**	**262 278 (225 685 to 303 507)**	**9·2 (4·6 to 14·3)**	**4 216 158 (3 589 877 to 4 949 994)**	**15·6 (13·7 to 17·4)**	**2 678 775 (2 274 031 to 3 178 849)**	**9·3 (5·0 to 14·2)**
Brunei	61 (50 to 75)	2·7 (−7·4 to 13·3)	1311 (1119 to 1530)	−4·1 (−7·1 to −0·8)	876 (723 to 1055)	0·8 (−8·7 to 10·0)
Japan	224 138 (194 703 to 258 848)	10·2 (6·8 to 13·6)	3 530 611 (2 999 793 to 4 177 416)	17·8 (15·7 to 19·8)	2 226 531 (1 894 705 to 2 641 329)	10·5 (7·5 to 13·2)
Singapore	2265 (1793 to 2805)	3·2 (−17·6 to 28·4)	37 905 (31 673 to 44 582)	11·5 (0·5 to 26·8)	24 746 (19 932 to 30 529)	6·3 (−12·5 to 28·8)
South Korea	35 814 (26 742 to 46 764)	−2·6 (−25·2 to 24·9)	646 331 (558 444 to 744 282)	−4·2 (−9·4 to 0·5)	426 622 (329 392 to 542 917)	−4·5 (−23·7 to 18·3)
**Western Europe**	**452 515 (389 889 to 523 953)**	**−7·4 (−10·8 to −3·7)**	**6 586 827 (5 634 206 to 7 629 868)**	**−8·1 (−10·9 to −5·3)**	**4 499 078 (3 822 323 to 5 279 978)**	**−8·6 (−12·0 to −5·3)**
Andorra	99 (78 to 123)	−3·2 (−21·4 to 23·7)	1253 (1060 to 1492)	−4·9 (−8·4 to −1·6)	901 (723 to 1116)	−4·4 (−20·4 to 17·7)
Austria	8270 (6966 to 9742)	−5·7 (−11·8 to 0·7)	126 914 (106 564 to 151 088)	−3·9 (−7·3 to −0·1)	84 853 (71 330 to 100 942)	−5·8 (−11·1 to −0·3)
Belgium	12 590 (10 464 to 15 119)	−7·3 (−17·1 to 1·9)	181 350 (153 218 to 215 482)	−9·2 (−15·4 to −3·8)	125 538 (104 406 to 150 465)	−8·8 (−17·2 to −0·8)
Cyprus	670 (564 to 795)	−8·1 (−14·9 to −0·6)	9644 (8181 to 11 464)	−1·7 (−4·6 to 1·5)	6844 (5754 to 8245)	−9·0 (−14·6 to −2·3)
Denmark	3984 (3264 to 4768)	−12·3 (−24·3 to −0·7)	55 336 (47 063 to 64 457)	−20·1 (−29·4 to −13·7)	39 831 (33 115 to 47 717)	−17·1 (−28·1 to −6·6)
Finland	6686 (5486 to 8082)	−2·3 (−11·1 to 6·7)	83 950 (70 832 to 99 790)	−7·4 (−13·6 to −2·7)	66 130 (54 299 to 81 192)	−5·0 (−13·0 to 2·6)
France	65 775 (55 179 to 78 174)	−0·6 (−8·5 to 8·0)	877 760 (739 227 to 1 050 674)	1·1 (−5·4 to 10·2)	611 185 (513 043 to 731 084)	−1·7 (−9·0 to 6·4)
Germany	77 634 (62 763 to 93 488)	−20·8 (−35·2 to −8·9)	1 201 668 (996 430 to 1 425 812)	−17·7 (−28·4 to −10·3)	823 713 (673 054 to 1 007 932)	−18·4 (−29·4 to −8·5)
Greece	12 568 (10 418 to 14 981)	−5·4 (−12·3 to 2·1)	192 563 (161 441 to 229 260)	−3·7 (−7·1 to −0·3)	128 762 (106 552 to 154 548)	−5·4 (−11·4 to 1·1)
Iceland	241 (199 to 287)	−0·1 (−8·8 to 9·0)	3373 (2842 to 4000)	−6·5 (−9·6 to −3·7)	2431 (2019 to 2932)	−2·9 (−10·1 to 4·7)
Ireland	2791 (2280 to 3337)	−5·0 (−15·1 to 6·9)	43 235 (36 676 to 51 258)	−4·9 (−8·2 to −1·0)	29 432 (24 290 to 35 763)	−6·0 (−14·3 to 4·2)
Israel	4730 (3860 to 5906)	−5·2 (−19·2 to 10·8)	69 596 (58 898 to 82 970)	−3·9 (−7·2 to −0·8)	46 920 (38 028 to 57 408)	−6·1 (−18·5 to 7·2)
Italy	90 981 (76 553 to 105 860)	−1·8 (−12·1 to 9·1)	1 370 308 (1 152 154 to 1 583 588)	−2·9 (−11·3 to 4·7)	898 329 (756 615 to 1 050 918)	−4·4 (−13·4 to 4·6)
Luxembourg	353 (290 to 421)	−6·6 (−16·8 to 3·6)	5022 (4319 to 5912)	−10·9 (−17·7 to −5·4)	3566 (2985 to 4264)	−9·7 (−18·6 to −0·6)
Malta	312 (250 to 384)	−4·0 (−18·5 to 10·2)	5145 (4332 to 6119)	−4·5 (−7·7 to −1·2)	3465 (2780 to 4309)	−5·0 (−16·8 to 7·1)
Netherlands	13 713 (11 747 to 15 989)	−14·2 (−22·3 to −5·3)	192 425 (169 675 to 221 880)	−16·8 (−24·8 to −8·7)	136 421 (116 347 to 160 138)	−15·9 (−23·7 to −7·2)
Norway	4777 (3969 to 5736)	−7·2 (−16·2 to 3·8)	67 207 (57 096 to 79 609)	−9·7 (−16·1 to −4·0)	46 646 (39 007 to 56 232)	−9·3 (−17·6 to 0·4)
Portugal	10 824 (9034 to 12 860)	−4·2 (−11·1 to 2·8)	166 660 (139 562 to 201 560)	−4·3 (−8·0 to −0·9)	113 147 (94 134 to 137 267)	−4·9 (−11·0 to 1·4)
Spain	57 098 (49 114 to 65 392)	−9·6 (−17·3 to −1·8)	830 915 (712 248 to 952 265)	−12·7 (−17·0 to −8·7)	561 948 (480 867 to 646 417)	−12·4 (−19·1 to −6·0)
Sweden	9658 (7866 to 11 723)	−0·4 (−10·5 to 11·4)	142 735 (121 220 to 169 371)	−4·0 (−9·0 to 0·4)	93 782 (77 008 to 113 348)	−2·7 (−11·0 to 6·4)
Switzerland	8234 (6373 to 10 456)	−1·7 (−19·8 to 19·9)	115 476 (97 869 to 138 074)	−6·1 (−10·6 to −2·2)	80 676 (63 754 to 100 552)	−5·0 (−20·1 to 12·8)
UK	60 525 (51 581 to 70 828)	−6·8 (−8·8 to −4·3)	838 693 (708 801 to 995 493)	−10·3 (−11·7 to −9·1)	593 711 (499 978 to 706 382)	−8·4 (−10·2 to −6·3)
**Southern Latin America**	**23 831 (19 455 to 28 671)**	**−3·5 (−10·7 to 5·0)**	**375 984 (315 602 to 448 261)**	**−4·4 (−7·0 to −2·0)**	**257 496 (211 125 to 311 373)**	**−4·6 (−10·7 to 2·5)**
Argentina	15 412 (12 605 to 18 722)	−2·7 (−10·5 to 5·7)	243 618 (205 012 to 290 358)	−4·0 (−7·1 to −0·7)	167 002 (136 040 to 202 061)	−3·6 (−10·2 to 2·9)
Chile	6508 (4983 to 8392)	−5·3 (−23·4 to 15·6)	104 523 (87 150 to 124 669)	−5·9 (−12·4 to 0·0)	70 944 (55 336 to 90 933)	−7·2 (−22·4 to 10·9)
Uruguay	1911 (1569 to 2289)	−5·2 (−12·4 to 2·5)	27 817 (23 177 to 33 439)	−4·6 (−7·4 to −1·7)	19 546 (16 151 to 23 493)	−6·1 (−11·7 to 0·4)
**Eastern Europe**	**84 368 (65 165 to 109 655)**	**−0·3 (−16·0 to 17·6)**	**1 554 081 (1 291 061 to 1 855 425)**	**−1·2 (−3·7 to 1·3)**	**1 037 741 (811 281 to 1 331 933)**	**−0·5 (−13·7 to 15·0)**
Belarus	4060 (3179 to 5018)	1·3 (−12·4 to 16·8)	72 664 (60 673 to 87 181)	−1·2 (−5·2 to 2·9)	48 733 (38 985 to 60 585)	0·4 (−11·3 to 13·3)
Estonia	805 (638 to 995)	−0·2 (−11·9 to 8·9)	13 540 (11 248 to 16 325)	−1·6 (−6·1 to 2·5)	9159 (7408 to 11 307)	−1·1 (−10·9 to 7·3)
Latvia	1202 (976 to 1472)	0·0 (−10·0 to 11·7)	20 677 (17 216 to 24 940)	−0·5 (−3·9 to 3·1)	13 994 (11 352 to 17 189)	−0·4 (−9·4 to 9·4)
Lithuania	1769 (1440 to 2170)	0·6 (−6·4 to 8·5)	30 147 (25 142 to 36 420)	−0·8 (−4·3 to 3·2)	20 304 (16 653 to 24 872)	0·1 (−6·0 to 6·9)
Moldova	1100 (905 to 1385)	−3·5 (−13·7 to 7·5)	20 777 (17 415 to 24 766)	−1·6 (−4·5 to 1·9)	13 823 (11 327 to 17 136)	−2·1 (−10·7 to 7·2)
Russia	55 562 (39 767 to 75 905)	0·0 (−23·1 to 27·2)	1 025 660 (849 865 to 1 229 568)	−1·0 (−4·8 to 3·1)	685 106 (508 315 to 920 093)	−0·3 (−20·1 to 23·7)
Ukraine	19 870 (15 171 to 26 624)	−1·3 (−18·1 to 18·8)	370 615 (306 917 to 442 613)	−1·6 (−4·8 to 2·2)	246 622 (191 357 to 320 476)	−1·3 (−16·0 to 16·1)
**Central Europe**	**59 119 (49 158 to 71 242)**	**−2·9 (−6·8 to 1·1)**	**1 033 615 (861 756 to 1 235 186)**	**−3·5 (−6·8 to −1·4)**	**696 428 (576 669 to 845 438)**	**−3·9 (−7·9 to −0·2)**
Albania	958 (772 to 1165)	−3·4 (−15·0 to 8·5)	18 048 (15 048 to 21 732)	−1·6 (−5·3 to 2·9)	11 979 (9651 to 14 810)	−3·0 (−12·4 to 7·3)
Bosnia and Herzegovina	1751 (1388 to 2195)	−2·2 (−16·5 to 13·8)	31 804 (26 614 to 37 994)	−0·5 (−4·0 to 3·5)	21 217 (17 002 to 26 502)	−2·1 (−14·1 to 11·1)
Bulgaria	4140 (3320 to 5247)	−1·4 (−12·4 to 10·5)	76 346 (63 003 to 92 366)	−0·6 (−4·8 to 2·8)	51 189 (41 554 to 64 039)	−1·7 (−11·9 to 8·8)
Croatia	2690 (2172 to 3335)	−0·3 (−12·7 to 12·2)	45 148 (37 772 to 53 815)	−1·3 (−4·9 to 2·3)	30 976 (25 056 to 38 427)	−0·9 (−11·1 to 9·3)
Czech Republic	5474 (4533 to 6592)	−2·6 (−8·8 to 4·1)	96 200 (80 221 to 115 100)	−1·4 (−4·9 to 2·6)	64 628 (53 720 to 78 313)	−3·3 (−8·7 to 2·4)
Hungary	5523 (4505 to 6773)	−1·0 (−10·4 to 8·8)	94 633 (78 970 to 113 490)	−1·6 (−5·7 to 2·0)	64 456 (52 914 to 79 536)	−1·4 (−9·8 to 7·4)
Macedonia	654 (543 to 798)	1·2 (−5·8 to 8·6)	12 787 (10 606 to 15 262)	0·3 (−3·4 to 4·0)	8600 (7095 to 10 502)	0·7 (−5·6 to 6·8)
Montenegro	248 (200 to 302)	1·4 (−10·4 to 12·7)	4553 (3772 to 5472)	0·4 (−3·0 to 3·8)	3040 (2460 to 3698)	1·3 (−8·7 to 11·4)
Poland	19 488 (15 936 to 23 766)	−6·6 (−15·8 to 3·2)	333 656 (277 898 to 399 034)	−8·8 (−18·1 to −3·0)	224 088 (184 403 to 272 346)	−8·7 (−17·5 to 0·2)
Romania	10 269 (8497 to 12 553)	−1·3 (−10·1 to 7·4)	182 066 (151 548 to 217 820)	−0·9 (−4·6 to 3·0)	122 264 (99 571 to 150 538)	−1·5 (−9·4 to 6·2)
Serbia	4305 (3582 to 5283)	1·8 (−6·1 to 11·1)	76 985 (64 237 to 91 993)	−0·4 (−3·8 to 3·5)	52 068 (43 092 to 63 732)	0·8 (−5·9 to 8·9)
Slovakia	2259 (1826 to 2804)	−0·6 (−10·3 to 11·1)	39 642 (33 110 to 47 216)	−1·3 (−4·9 to 2·8)	27 138 (22 050 to 33 781)	−1·4 (−10·1 to 8·4)
Slovenia	1359 (1095 to 1661)	−4·6 (−16·0 to 7·7)	21 745 (18 129 to 26 165)	−2·4 (−5·5 to 1·3)	14 785 (11 857 to 18 020)	−5·1 (−14·7 to 5·1)
**Central Asia**	**12 669 (10 564 to 15 413)**	**−1·0 (−5·5 to 3·9)**	**247 867 (207 340 to 294 247)**	**−0·9 (−2·6 to 0·9)**	**162 755 (135 702 to 197 877)**	**−1·3 (−5·4 to 3·0)**
Armenia	1060 (876 to 1307)	3·6 (−6·8 to 14·7)	18 476 (15 412 to 22 206)	−0·5 (−3·9 to 3·0)	12 426 (10 313 to 15 383)	2·0 (−6·8 to 11·3)
Azerbaijan	1506 (1197 to 1883)	1·9 (−10·2 to 17·7)	30 747 (25 808 to 36 580)	0·4 (−3·1 to 3·9)	20 191 (16 307 to 25 036)	1·0 (−9·7 to 14·8)
Georgia	1806 (1461 to 2234)	2·2 (−13·4 to 16·3)	30 310 (25 251 to 36 053)	0·2 (−3·7 to 4·3)	20 639 (16 866 to 25 643)	1·7 (−11·3 to 14·1)
Kazakhstan	2603 (2097 to 3358)	−4·2 (−17·6 to 10·7)	56 927 (47 459 to 68 027)	−0·8 (−4·7 to 2·7)	35 883 (28 877 to 45 860)	−3·9 (−15·5 to 8·7)
Kyrgyzstan	677 (557 to 816)	−0·2 (−7·9 to 8·1)	13 141 (11 032 to 15 644)	−0·2 (−4·2 to 3·6)	8702 (7189 to 10 576)	0·2 (−6·7 to 7·3)
Mongolia	265 (213 to 327)	−1·8 (−13·6 to 10·9)	5635 (4703 to 6648)	−0·2 (−3·6 to 3·2)	3735 (2989 to 4546)	−3·3 (−13·4 to 8·0)
Tajikistan	655 (535 to 806)	−2·2 (−12·3 to 11·0)	12 872 (10 841 to 15 131)	−4·2 (−7·3 to −0·7)	8596 (7103 to 10 510)	−2·8 (−11·8 to 9·1)
Turkmenistan	506 (424 to 608)	−4·6 (−10·8 to 2·3)	10 455 (8785 to 12 366)	−2·3 (−5·6 to 1·1)	6766 (5636 to 8176)	−4·3 (−9·7 to 1·4)
Uzbekistan	3591 (2942 to 4390)	−1·3 (−9·7 to 7·9)	69 303 (57 761 to 81 697)	−0·9 (−4·5 to 2·5)	45 817 (37 701 to 56 067)	−1·3 (−8·5 to 6·4)
**Central Latin America**	**67 443 (56 996 to 80 037)**	**−7·2 (−10·0 to −4·2)**	**1 268 251 (1 073 553 to 1 503 468)**	**−5·2 (−6·5 to −4·2)**	**819 513 (685 355 to 989 762)**	**−6·9 (−9·3 to −4·4)**
Colombia	12 384 (10 275 to 14 879)	−7·3 (−14·6 to 0·9)	248 583 (210 857 to 295 501)	−4·9 (−7·8 to −1·8)	158 120 (130 223 to 189 645)	−6·8 (−13·1 to 0·3)
Costa Rica	1687 (1417 to 2012)	−6·0 (−13·7 to 1·5)	31 865 (26 781 to 37 969)	−4·2 (−7·3 to −0·7)	20 526 (17 088 to 24 631)	−5·8 (−12·3 to 0·4)
El Salvador	2124 (1761 to 2563)	−6·3 (−15·6 to 3·3)	37 416 (31 301 to 44 669)	−3·7 (−7·4 to −0·5)	25 185 (20 658 to 30 567)	−7·0 (−14·8 to 1·0)
Guatemala	3052 (2304 to 3929)	−2·6 (−20·9 to 18·0)	57 458 (48 152 to 67 876)	−2·6 (−5·8 to 0·7)	38 267 (29 854 to 48 962)	−3·8 (−19·7 to 14·2)
Honduras	1847 (1418 to 2291)	−2·0 (−19·8 to 17·0)	30 015 (25 259 to 35 678)	−4·3 (−7·5 to −1·3)	22 506 (17 410 to 28 506)	−3·6 (−19·0 to 14·0)
Mexico	37 128 (31 423 to 44 041)	−6·2 (−9·4 to −3·1)	679 581 (571 934 to 806 887)	−3·9 (−5·0 to −3·0)	437 563 (367 299 to 525 198)	−5·5 (−7·9 to −3·2)
Nicaragua	1390 (1136 to 1696)	−5·0 (−17·2 to 8·3)	25 410 (21 451 to 30 239)	−3·9 (−7·4 to −0·5)	16 428 (13 353 to 20 057)	−4·8 (−15·2 to 6·8)
Panama	1227 (1014 to 1507)	−8·2 (−18·8 to 3·7)	22 348 (18 867 to 26 700)	−6·6 (−9·9 to −3·2)	14 478 (11 989 to 17 779)	−8·3 (−17·6 to 2·0)
Venezuela	6605 (5342 to 8050)	−15·1 (−25·9 to −1·7)	135 574 (115 292 to 158 925)	−13·4 (−20·5 to −8·6)	86 441 (69 422 to 106 289)	−15·4 (−24·7 to −3·2)
**Andean Latin America**	**11 513 (9504 to 14 155)**	**−3·2 (−12·3 to 6·9)**	**201 807 (171 097 to 237 657)**	**−6·0 (−8·5 to −3·6)**	**137 206 (113 336 to 168 798)**	**−4·5 (−12·0 to 3·7)**
Bolivia	2285 (1759 to 2899)	2·9 (−11·7 to 21·3)	38 118 (31 800 to 45 400)	−3·5 (−6·2 to −0·5)	27 439 (21 628 to 34 385)	0·5 (−12·8 to 16·8)
Ecuador	3023 (2500 to 3608)	−6·3 (−13·2 to 1·1)	55 865 (47 213 to 65 937)	−5·1 (−8·3 to −1·8)	36 711 (30 333 to 44 613)	−7·0 (−13·0 to −0·7)
Peru	6205 (4847 to 7733)	−4·0 (−19·2 to 12·8)	107 824 (91 186 to 126 723)	−7·5 (−11·6 to −3·4)	73 056 (58 539 to 90 994)	−5·1 (−17·5 to 9·2)
**Caribbean**	**13 624 (11 487 to 16 276)**	**−3·5 (−8·4 to 1·6)**	**244 819 (206 229 to 289 536)**	**−4·9 (−6·5 to −3·2)**	**161 853 (135 793 to 197 434)**	**−4·4 (−8·6 to −0·1)**
Antigua and Barbuda	21 (17 to 25)	0·6 (−10·8 to 13·4)	372 (312 to 439)	−1·6 (−5·1 to 1·8)	252 (206 to 307)	−0·5 (−10·1 to 10·8)
The Bahamas	88 (71 to 108)	4·1 (−6·6 to 15·8)	1688 (1413 to 2012)	0·5 (−2·5 to 4·4)	1162 (944 to 1420)	2·1 (−6·8 to 12·0)
Barbados	136 (111 to 164)	5·7 (−4·2 to 16·1)	2224 (1869 to 2641)	−2·0 (−5·3 to 1·7)	1557 (1284 to 1890)	2·7 (−5·4 to 11·5)
Belize	37 (30 to 46)	4·6 (−7·5 to 17·1)	707 (599 to 836)	−3·9 (−7·1 to −0·7)	505 (419 to 609)	2·7 (−8·8 to 13·8)
Bermuda	21 (17 to 26)	−1·0 (−14·8 to 14·1)	356 (301 to 423)	−4·0 (−7·3 to −0·8)	249 (201 to 309)	−3·2 (−14·3 to 9·0)
Cuba	6149 (5118 to 7370)	−3·2 (−11·7 to 6·0)	103 227 (86 496 to 122 320)	−4·8 (−7·9 to −1·4)	70 115 (58 080 to 84 845)	−4·3 (−11·3 to 3·1)
Dominica	21 (17 to 26)	0·7 (−9·7 to 13·5)	357 (299 to 427)	−3·5 (−6·9 to −0·4)	246 (201 to 301)	−1·8 (−10·6 to 9·2)
Dominican Republic	2233 (1796 to 2743)	−7·6 (−20·4 to 5·4)	39 760 (33 575 to 47 321)	−6·2 (−9·4 to −3·1)	26 694 (21 595 to 32 836)	−8·0 (−19·0 to 3·2)
Grenada	28 (23 to 34)	5·1 (−7·0 to 16·9)	449 (375 to 534)	−2·7 (−5·8 to 0·5)	319 (261 to 390)	2·8 (−7·4 to 13·0)
Guyana	80 (66 to 96)	2·5 (−7·0 to 12·5)	1850 (1570 to 2186)	−1·6 (−5·2 to 1·5)	1221 (1002 to 1480)	0·1 (−7·8 to 8·9)
Haiti	1192 (931 to 1499)	1·7 (−11·4 to 15·8)	24 561 (20 588 to 29 210)	−6·3 (−9·5 to −2·6)	17 213 (13 710 to 21 591)	−0·7 (−12·0 to 11·3)
Jamaica	951 (773 to 1166)	1·5 (−12·2 to 16·2)	15 075 (12 743 to 17 797)	−4·3 (−7·6 to −1·3)	10 501 (8566 to 12 957)	0·1 (−11·6 to 12·3)
Puerto Rico	2081 (1686 to 2535)	1·3 (−7·3 to 10·8)	31 203 (26 216 to 37 065)	−2·9 (−6·1 to 0·3)	22 554 (18 612 to 27 815)	−0·8 (−8·3 to 7·2)
Saint Lucia	57 (47 to 69)	1·2 (−6·3 to 9·0)	975 (811 to 1156)	−2·1 (−5·5 to 1·2)	672 (553 to 817)	0·1 (−6·2 to 6·7)
Saint Vincent and the Grenadines	24 (20 to 29)	−5·8 (−14·2 to 3·1)	454 (383 to 539)	−4·6 (−7·5 to −1·6)	307 (255 to 373)	−5·6 (−12·8 to 2·2)
Suriname	118 (98 to 143)	7·3 (−1·3 to 16·8)	2097 (1764 to 2484)	−1·6 (−4·3 to 1·5)	1487 (1225 to 1797)	3·6 (−3·9 to 11·3)
Trinidad and Tobago	340 (283 to 410)	−3·3 (−10·7 to 4·9)	6739 (5667 to 7967)	−2·9 (−6·1 to 0·4)	4441 (3665 to 5432)	−4·2 (−10·4 to 2·8)
Virgin Islands	48 (39 to 58)	−5·6 (−13·7 to 5·0)	881 (737 to 1051)	−4·0 (−7·2 to −0·5)	613 (501 to 751)	−6·2 (−13·8 to 2·9)
**Tropical Latin America**	**82 383 (70 588 to 97 167)**	**0·1 (−3·7 to 4·0)**	**1 725 736 (1 470 749 to 2 025 314)**	**5·0 (2·4 to 7·9)**	**1 104 940 (931 687 to 1 314 278)**	**3·1 (−0·3 to 6·7)**
Brazil	80 600 (69 174 to 94 940)	0·1 (−3·7 to 4·0)	1 691 024 (1 440 967 to 1 983 529)	5·2 (2·5 to 8·2)	1 081 903 (911 837 to 1 287 687)	3·2 (−0·2 to 6·8)
Paraguay	1783 (1467 to 2186)	−2·0 (−12·6 to 8·8)	34 712 (29 060 to 41 134)	−6·1 (−8·9 to −3·0)	23 037 (18 916 to 28 040)	−3·3 (−12·3 to 5·9)
**East Asia**	**495 128 (423 608 to 582 787)**	**−0·5 (−9·5 to 4·3)**	**10 767 181 (9 212 998 to 12 591 276)**	**5·4 (4·2 to 6·7)**	**6 866 579 (5 812 325 to 8 086 374)**	**0·2 (−6·6 to 4·0)**
China	476 898 (407 695 to 560 628)	−0·1 (−9·5 to 4·9)	10 427 487 (8 917 543 to 12 196 329)	5·6 (4·4 to 6·9)	6 637 268 (5 606 035 to 7 806 164)	0·5 (−6·5 to 4·4)
North Korea	6098 (5062 to 7511)	3·4 (−8·5 to 19·6)	136 430 (114 541 to 161 576)	−3·8 (−7·0 to −0·4)	90 179 (74 251 to 108 648)	2·5 (−7·5 to 15·7)
Taiwan (province of China)	12 132 (9878 to 14 776)	−14·6 (−26·3 to 0·6)	203 264 (171 769 to 238 879)	11·1 (3·6 to 23·8)	139 132 (114 655 to 168 804)	−9·7 (−20·8 to 4·3)
**Southeast Asia**	**168 498 (143 918 to 200 845)**	**6·5 (0·3 to 14·6)**	**3 337 721 (2 835 683 to 3 942 605)**	**−1·5 (−2·6 to −0·4)**	**2 295 779 (1 935 096 to 2 717 204)**	**3·8 (−1·0 to 9·5)**
Cambodia	2439 (2073 to 2955)	17·8 (4·0 to 44·7)	50 393 (42 605 to 59 560)	−3·9 (−7·3 to −0·6)	37 733 (31 661 to 45 301)	11·4 (0·8 to 30·8)
Indonesia	45 591 (38 307 to 54 896)	32·6 (17·1 to 54·6)	1 111 081 (942 834 to 1 320 195)	−0·1 (−1·3 to 1·1)	703 600 (581 417 to 843 705)	20·6 (10·9 to 33·8)
Laos	919 (760 to 1126)	15·5 (2·7 to 33·4)	20 678 (17 491 to 24 443)	−1·5 (−5·1 to 2·1)	13 805 (11 264 to 16 642)	10·2 (0·6 to 22·7)
Malaysia	6579 (5517 to 7893)	−7·6 (−14·1 to 0·7)	159 491 (135 859 to 189 529)	−0·9 (−4·8 to 3·3)	100 999 (84 056 to 121 357)	−6·8 (−12·5 to 0·1)
Maldives	70 (55 to 87)	1·2 (−14·0 to 21·6)	1464 (1236 to 1731)	0·5 (−2·5 to 4·0)	948 (761 to 1166)	1·2 (−12·1 to 19·4)
Mauritius	462 (382 to 559)	−6·0 (−15·9 to 5·6)	9941 (8400 to 11 689)	−3·1 (−6·7 to 0·2)	6282 (5196 to 7502)	−6·5 (−14·9 to 3·3)
Myanmar	17 857 (15 099 to 21 127)	8·8 (−2·3 to 22·6)	232 112 (198 607 to 272 903)	−3·1 (−6·4 to 0·2)	240 489 (199 922 to 286 798)	4·5 (−5·0 to 15·8)
Philippines	14 942 (12 213 to 18 279)	1·3 (−10·3 to 13·4)	359 689 (304 824 to 426 756)	−0·8 (−3·8 to 2·3)	230 296 (187 155 to 280 412)	0·6 (−9·5 to 11·0)
Sri Lanka	6680 (5277 to 8346)	−10·4 (−26·6 to 7·1)	127 331 (107 863 to 149 887)	−0·1 (−3·5 to 3·9)	86 779 (69 960 to 107 916)	−7·4 (−22·0 to 8·2)
Seychelles	36 (30 to 43)	−2·4 (−10·7 to 6·4)	653 (551 to 769)	−1·1 (−3·9 to 2·0)	439 (364 to 526)	−3·0 (−10·2 to 4·9)
Thailand	30 293 (24 884 to 37 126)	−5·2 (−15·9 to 7·6)	597 698 (505 189 to 703 496)	−1·5 (−5·0 to 2·2)	395 408 (325 461 to 482 250)	−5·2 (−13·9 to 4·9)
Timor-Leste	172 (134 to 219)	16·0 (−4·9 to 51·2)	3976 (3338 to 4745)	−1·2 (−4·5 to 2·1)	2761 (2165 to 3458)	11·5 (−6·4 to 38·4)
Vietnam	42 459 (35 595 to 50 802)	1·8 (−10·9 to 17·8)	655 099 (558 892 to 775 483)	−2·6 (−6·0 to 0·7)	475 067 (398 602 to 568 773)	−0·4 (−11·3 to 13·1)
**Oceania**	**968 (794 to 1187)**	**−7·3 (−14·5 to 0·8)**	**28 064 (23 704 to 33 276)**	**−1·6 (−3·8 to 0·2)**	**16 322 (13 394 to 19 833)**	**−5·9 (−12·6 to 1·3)**
American Samoa	8 (6 to 9)	−5·0 (−17·5 to 8·9)	173 (147 to 204)	1·0 (−2·7 to 4·7)	114 (92 to 138)	−4·5 (−15·4 to 8·2)
Federated States of Micronesia	18 (14 to 22)	−2·6 (−15·4 to 13·3)	311 (263 to 364)	−1·0 (−4·7 to 2·8)	251 (197 to 313)	−3·3 (−16·1 to 12·8)
Fiji	127 (99 to 163)	−7·2 (−24·3 to 13·5)	3182 (2676 to 3767)	−0·7 (−4·0 to 3·1)	2049 (1629 to 2575)	−6·3 (−22·3 to 12·2)
Guam	54 (44 to 66)	−6·1 (−15·3 to 4·4)	1061 (890 to 1259)	−1·8 (−5·1 to 1·8)	721 (588 to 871)	−5·5 (−13·9 to 3·8)
Kiribati	18 (15 to 23)	13·8 (−0·5 to 31·0)	298 (253 to 352)	−1·2 (−4·4 to 1·7)	269 (220 to 333)	9·1 (−3·7 to 24·1)
Marshall Islands	5 (4 to 7)	−15·9 (−25·5 to −5·2)	159 (134 to 189)	−1·7 (−5·0 to 1·8)	92 (74 to 115)	−13·8 (−22·4 to −4·0)
Northern Mariana Islands	6 (5 to 7)	−4·3 (−19·5 to 15·7)	129 (111 to 149)	−1·9 (−5·2 to 1·9)	85 (70 to 105)	−5·1 (−18·5 to 12·0)
Papua New Guinea	555 (439 to 701)	1·6 (−11·1 to 16·3)	15 398 (13 008 to 18 206)	−2·2 (−5·7 to 1·0)	9665 (7658 to 11 921)	−0·7 (−11·4 to 11·8)
Samoa	50 (41 to 62)	−4·4 (−15·9 to 9·5)	784 (662 to 930)	−1·7 (−5·0 to 1·3)	628 (518 to 770)	−4·4 (−15·3 to 8·6)
Solomon Islands	63 (52 to 78)	3·4 (−7·1 to 17·2)	1280 (1081 to 1514)	−0·5 (−3·8 to 2·5)	974 (791 to 1195)	1·2 (−8·7 to 14·6)
Tonga	31 (26 to 38)	0·8 (−12·9 to 16·9)	499 (420 to 593)	0·0 (−3·0 to 3·6)	374 (308 to 460)	−0·1 (−12·8 to 14·5)
Vanuatu	33 (27 to 42)	2·5 (−8·8 to 15·2)	708 (594 to 843)	−1·1 (−4·3 to 2·5)	530 (429 to 669)	1·2 (−9·6 to 13·4)
**North Africa and Middle East**	**120 035 (102 120 to 141 652)**	**−1·8 (−7·6 to 4·8)**	**2 613 225 (2 220 174 to 3 075 460)**	**−1·0 (−2·7 to 1·5)**	**1 669 040 (1 404 463 to 1 982 410)**	**−1·3 (−6·2 to 4·0)**
Afghanistan	2903 (2360 to 3584)	7·1 (−2·9 to 22·4)	65 639 (54 920 to 77 796)	−1·5 (−4·8 to 2·5)	48 654 (39 657 to 59 945)	5·1 (−4·1 to 17·0)
Algeria	11 868 (9916 to 14 314)	−1·8 (−10·9 to 8·0)	223 164 (188 279 to 265 420)	−4·3 (−7·4 to −1·0)	156 627 (130 028 to 189 001)	−2·4 (−10·1 to 6·7)
Bahrain	128 (102 to 160)	−1·1 (−16·2 to 15·6)	3091 (2638 to 3618)	−2·1 (−5·1 to 1·0)	1983 (1576 to 2469)	−3·4 (−16·5 to 11·2)
Egypt	14 929 (12 172 to 18 150)	−1·5 (−12·1 to 10·7)	355 367 (306 335 to 409 862)	0·9 (−3·0 to 5·4)	218 670 (181 299 to 263 821)	−0·8 (−10·3 to 9·7)
Iran	16 739 (13 611 to 20 490)	3·7 (−10·5 to 21·9)	368 528 (311 492 to 437 404)	−1·2 (−4·2 to 2·0)	235 690 (189 845 to 288 441)	2·4 (−10·5 to 18·8)
Iraq	4294 (3433 to 5344)	−1·6 (−16·4 to 13·4)	99 821 (84 081 to 119 541)	−1·4 (−4·8 to 2·2)	65 480 (51 944 to 80 425)	−1·8 (−15·9 to 12·4)
Jordan	1106 (868 to 1416)	−3·8 (−20·5 to 16·4)	25 413 (21 281 to 30 183)	−0·8 (−4·6 to 2·7)	16 197 (12 628 to 20 464)	−3·5 (−19·2 to 15·0)
Kuwait	265 (197 to 344)	0·1 (−20·8 to 25·1)	7097 (6063 to 8425)	−2·5 (−5·6 to 0·3)	4411 (3363 to 5676)	−0·6 (−18·5 to 21·5)
Lebanon	2071 (1704 to 2531)	−12·5 (−25·6 to 3·4)	45 656 (37 962 to 54 771)	−3·0 (−6·5 to 0·4)	27 451 (22 314 to 33 342)	−12·6 (−24·2 to 2·0)
Libya	1313 (1096 to 1585)	−0·3 (−10·9 to 10·5)	25 567 (21 548 to 30 392)	−0·7 (−3·8 to 2·6)	17 978 (14 741 to 21 844)	−1·2 (−10·8 to 8·9)
Morocco	9169 (7460 to 11 204)	7·6 (−6·5 to 38·1)	197 480 (165 612 to 233 425)	−4·3 (−7·6 to −0·8)	126 157 (103 001 to 153 832)	4·5 (−6·9 to 24·9)
Oman	439 (374 to 526)	−7·5 (−17·3 to 3·8)	10 353 (8822 to 12 263)	−4·9 (−8·2 to −1·2)	6503 (5393 to 7783)	−8·7 (−17·2 to 0·9)
Palestine	497 (414 to 605)	−2·7 (−13·7 to 10·0)	11 635 (9767 to 13 847)	−1·6 (−5·3 to 2·3)	7619 (6286 to 9256)	−2·3 (−12·4 to 8·5)
Qatar	93 (68 to 124)	−4·2 (−26·7 to 22·0)	2706 (2292 to 3174)	−2·7 (−5·6 to 0·5)	1682 (1255 to 2220)	−4·8 (−23·7 to 19·8)
Saudi Arabia	4148 (3515 to 4980)	3·8 (−9·6 to 21·2)	91 232 (77 702 to 107 276)	−0·2 (−1·6 to 1·0)	58 495 (48 953 to 69 907)	2·5 (−8·8 to 16·4)
Sudan	4164 (3425 to 5077)	8·1 (−0·5 to 21·5)	99 360 (83 462 to 118 168)	−2·8 (−6·1 to 1·0)	63 559 (52 164 to 77 052)	5·0 (−2·2 to 14·5)
Syria	3132 (2608 to 3727)	−0·6 (−7·9 to 7·1)	68 793 (58 115 to 81 155)	−2·5 (−6·4 to 0·9)	44 016 (36 492 to 53 053)	−1·2 (−8·5 to 6·0)
Tunisia	4558 (3681 to 5599)	−1·9 (−16·9 to 12·3)	85 220 (71 552 to 101 268)	−2·3 (−5·4 to 0·8)	58 122 (47 222 to 71 312)	−2·3 (−15·6 to 10·2)
Turkey	35 355 (28 754 to 43 599)	−7·2 (−21·2 to 10·2)	754 169 (639 274 to 887 565)	2·5 (−2·4 to 9·9)	462 429 (375 893 to 562 128)	−4·1 (−16·5 to 11·2)
United Arab Emirates	318 (261 to 389)	1·9 (−14·1 to 21·2)	10 301 (8769 to 12 096)	−4·3 (−7·6 to −1·0)	6303 (5108 to 7835)	0·2 (−14·7 to 17·9)
Yemen	2547 (2088 to 3117)	5·7 (−5·6 to 20·8)	61 063 (51 634 to 72 314)	−4·5 (−7·7 to −0·7)	40 803 (33 454 to 49 862)	2·9 (−7·0 to 15·3)
**South Asia**	**178 664 (147 248 to 221 989)**	**16·6 (5·5 to 28·6)**	**3 747 443 (3 149 228 to 4 457 380)**	**−4·6 (−5·4 to −3·8)**	**2 494 935 (2 063 839 to 3 062 413)**	**9·1 (1·8 to 16·6)**
Bangladesh	16 463 (13 583 to 20 470)	−25·9 (−36·2 to −13·2)	401 328 (337 842 to 477 034)	−0·6 (−3·9 to 3·3)	229 138 (187 502 to 282 360)	−22·6 (−31·5 to −12·3)
Bhutan	100 (80 to 123)	5·4 (−10·8 to 26·8)	1712 (1435 to 2023)	−2·8 (−5·7 to 1·1)	1249 (1020 to 1542)	1·5 (−11·6 to 17·4)
India	142 927 (117 019 to 177 846)	23·4 (10·2 to 36·5)	2 933 814 (2 466 582 to 3 493 076)	−5·5 (−6·2 to −4·7)	1 996 152 (1 642 642 to 2 451 329)	13·5 (5·5 to 21·9)
Nepal	3294 (2678 to 4084)	27·0 (3·6 to 57·2)	64 140 (53 603 to 76 362)	−5·4 (−8·6 to −2·0)	46 390 (37 691 to 56 890)	14·8 (−0·5 to 34·5)
Pakistan	15 880 (12 814 to 19 871)	10·7 (−6·2 to 38·4)	346 449 (290 105 to 410 068)	−4·3 (−7·4 to −1·3)	222 007 (179 406 to 273 549)	5·8 (−7·1 to 24·5)
**Southern sub-Saharan Africa**	**10 893 (9030 to 13 274)**	**9·9 (0·9 to 18·8)**	**205 047 (172 093 to 245 564)**	**−3·0 (−4·2 to −1·7)**	**141 999 (117 766 to 173 195)**	**6·4 (−0·6 to 13·6)**
Botswana	189 (94 to 267)	2·2 (−42·7 to 36·3)	3896 (3270 to 4611)	−3·2 (−7·0 to 0·7)	2759 (1540 to 3845)	−0·5 (−38·2 to 30·2)
eSwatini	86 (55 to 123)	−7·5 (−31·7 to 18·6)	1987 (1659 to 2369)	−4·8 (−8·0 to −1·3)	1316 (884 to 1842)	−8·6 (−29·7 to 15·0)
Lesotho	214 (157 to 278)	0·7 (−21·1 to 23·9)	4363 (3630 to 5212)	−1·7 (−5·2 to 2·4)	2965 (2209 to 3875)	−0·9 (−20·9 to 21·1)
Namibia	226 (140 to 305)	2·2 (−29·3 to 26·7)	4174 (3493 to 4971)	−3·4 (−6·6 to −0·2)	3186 (2093 to 4263)	−0·7 (−27·9 to 21·6)
South Africa	8545 (7 118 to 10 394)	6·4 (−2·4 to 15·2)	167 424 (140 580 to 200 571)	−3·1 (−4·4 to −1·8)	110 748 (92 297 to 134 519)	3·4 (−3·2 to 10·5)
Zimbabwe	1634 (1262 to 2091)	40·1 (17·4 to 97·7)	23 203 (19 358 to 27 775)	−2·5 (−5·9 to 1·1)	21 025 (16 364 to 26 711)	31·2 (10·6 to 76·1)
**Western sub-Saharan Africa**	**19 288 (15 849 to 23 579)**	**3·7 (−3·7 to 11·3)**	**363 434 (308 212 to 428 346)**	**−2·7 (−4·8 to 0·3)**	**286 143 (234 972 to 347 258)**	**1·8 (−4·8 to 8·4)**
Benin	622 (506 to 769)	−4·5 (−14·4 to 7·1)	11 021 (9397 to 12 958)	−7·1 (−11·8 to −2·7)	9361 (7747 to 11 453)	−5·0 (−14·2 to 5·7)
Burkina Faso	884 (722 to 1104)	−3·6 (−13·7 to 9·6)	15 600 (13 128 to 18 543)	−3·9 (−7·0 to −0·4)	13 124 (10 715 to 16 097)	−5·2 (−14·2 to 6·4)
Cameroon	1811 (1405 to 2332)	−0·2 (−14·4 to 15·0)	26 814 (22 544 to 31 740)	−4·2 (−7·4 to −1·0)	24 206 (18 665 to 31 079)	−1·4 (−15·4 to 13·4)
Cape Verde	74 (60 to 91)	4·4 (−6·6 to 15·4)	1185 (990 to 1417)	−3·8 (−7·2 to 0·0)	866 (705 to 1067)	1·7 (−7·8 to 11·3)
Chad	723 (588 to 896)	−4·2 (−13·4 to 5·8)	12 784 (10 755 to 15 100)	−4·0 (−6·8 to −1·1)	10 307 (8349 to 12 656)	−4·8 (−13·7 to 4·9)
Côte d'Ivoire	1287 (1033 to 1596)	3·1 (−6·0 to 13·3)	22 894 (19 142 to 27 192)	−3·1 (−6·6 to 0·3)	19 796 (15 952 to 24 347)	2·2 (−6·4 to 11·7)
The Gambia	99 (80 to 124)	4·4 (−8·5 to 18·9)	1696 (1432 to 2011)	−3·7 (−6·9 to −0·1)	1428 (1150 to 1763)	2·6 (−9·9 to 15·4)
Ghana	1990 (1599 to 2437)	12·0 (−1·1 to 29·4)	32 390 (27 243 to 38 466)	−3·0 (−6·4 to 0·2)	27 517 (22 421 to 33 454)	8·7 (−3·1 to 23·4)
Guinea	765 (615 to 957)	1·5 (−11·3 to 13·9)	13 260 (11 180 to 15 706)	−4·4 (−7·2 to −1·2)	11 402 (9270 to 14 231)	0·2 (−11·9 to 11·3)
Guinea-Bissau	109 (88 to 133)	−2·2 (−11·8 to 10·5)	1993 (1679 to 2360)	−4·4 (−7·6 to −1·2)	1681 (1352 to 2074)	−3·4 (−12·9 to 8·6)
Liberia	249 (202 to 307)	3·9 (−5·7 to 16·1)	4642 (3907 to 5539)	−3·5 (−7·2 to 0·1)	3766 (3060 to 4614)	2·3 (−6·0 to 12·5)
Mali	924 (733 to 1179)	4·6 (−7·8 to 20·6)	15 724 (13 238 to 18 678)	−3·7 (−6·8 to 0·0)	12 949 (10 224 to 16 284)	1·4 (−10·5 to 16·5)
Mauritania	291 (225 to 372)	3·0 (−12·6 to 19·6)	4793 (4035 to 5686)	−3·0 (−6·0 to 0·5)	4113 (3235 to 5295)	0·4 (−14·1 to 15·0)
Niger	868 (684 to 1103)	1·9 (−9·1 to 16·5)	17 365 (14 617 to 20 614)	−2·8 (−6·0 to 0·5)	13 730 (10 676 to 17 267)	0·9 (−9·7 to 15·1)
Nigeria	6911 (5333 to 8763)	6·0 (−10·8 to 22·0)	152 733 (129 008 to 179 828)	−0·9 (−5·7 to 6·9)	107 197 (83 511 to 134 751)	3·8 (−11·7 to 18·3)
São Tomé and Príncipe	16 (13 to 21)	3·2 (−9·8 to 17·2)	259 (217 to 311)	−3·5 (−6·6 to 0·2)	199 (159 to 247)	1·4 (−10·0 to 13·2)
Senegal	1043 (856 to 1284)	6·4 (−2·4 to 16·0)	15 915 (13 412 to 18 930)	−6·0 (−9·2 to −2·7)	14 552 (11 928 to 17 884)	3·5 (−3·9 to 11·6)
Sierra Leone	246 (196 to 305)	−0·3 (−10·9 to 12·6)	5612 (4710 to 6643)	−3·6 (−6·5 to −0·3)	4222 (3353 to 5182)	−0·7 (−10·5 to 10·4)
Togo	376 (303 to 463)	−1·7 (−12·6 to 10·0)	6730 (5653 to 8034)	−4·1 (−7·6 to −0·9)	5723 (4601 to 7009)	−2·3 (−12·1 to 8·5)
**Eastern sub-Saharan Africa**	**29 557 (24 407 to 36 841)**	**6·5 (0·1 to 14·7)**	**508 756 (428 178 to 603 846)**	**−4·1 (−5·4 to −2·8)**	**406 808 (336 539 to 502 313)**	**3·5 (−1·6 to 9·7)**
Burundi	639 (511 to 820)	4·2 (−7·4 to 16·0)	12 408 (10 409 to 14 752)	−4·4 (−7·5 to −0·7)	9278 (7425 to 11 594)	0·9 (−9·7 to 12·0)
Comoros	58 (47 to 72)	4·0 (−8·9 to 17·4)	993 (832 to 1172)	−4·1 (−7·9 to −0·7)	807 (657 to 990)	1·3 (−10·6 to 13·3)
Djibouti	98 (74 to 125)	6·4 (−12·2 to 24·6)	1639 (1378 to 1947)	−4·1 (−6·9 to −1·2)	1325 (1010 to 1679)	3·3 (−13·8 to 19·9)
Eritrea	296 (235 to 373)	9·4 (−3·2 to 21·7)	5465 (4580 to 6486)	−5·5 (−9·0 to −1·9)	4487 (3587 to 5602)	4·4 (−5·8 to 15·7)
Ethiopia	8664 (6923 to 10 914)	9·3 (−3·9 to 26·1)	145 109 (121 512 to 172 680)	−4·1 (−7·7 to −0·5)	120 681 (95 728 to 151 710)	6·0 (−5·2 to 20·2)
Kenya	3205 (2448 to 4091)	15·3 (2·0 to 43·5)	61 120 (51 624 to 72 287)	−2·1 (−2·8 to −1·5)	42 739 (33 585 to 53 535)	9·6 (−0·4 to 26·2)
Madagascar	1486 (1147 to 1876)	−1·4 (−16·2 to 12·0)	31 885 (26 736 to 37 901)	−3·1 (−6·8 to 0·3)	21 798 (17 049 to 27 350)	−1·2 (−14·7 to 11·6)
Malawi	1509 (1143 to 1970)	4·3 (−15·0 to 27·7)	24 419 (20 280 to 29 218)	−3·4 (−6·9 to 0·0)	20 730 (15 694 to 26 556)	1·9 (−16·2 to 22·4)
Mozambique	2554 (1995 to 3300)	−2·6 (−16·4 to 14·8)	41 436 (34 782 to 49 571)	−4·0 (−7·4 to −0·7)	34 956 (27 433 to 44 059)	−4·1 (−16·8 to 11·5)
Rwanda	1004 (799 to 1259)	16·2 (1·5 to 31·9)	15 536 (13 147 to 18 296)	−4·1 (−7·5 to −0·3)	12 682 (10 253 to 15 694)	11·0 (−2·2 to 25·7)
Somalia	680 (536 to 864)	−0·4 (−10·0 to 12·5)	12 957 (10 830 to 15 461)	−5·2 (−8·7 to −1·7)	10 031 (7993 to 12 698)	−1·7 (−11·2 to 10·3)
South Sudan	881 (677 to 1138)	10·2 (−6·9 to 28·6)	18 205 (15 254 to 21 613)	−4·9 (−8·5 to −1·6)	13 002 (10 053 to 16 543)	7·0 (−8·2 to 24·7)
Tanzania	4728 (3807 to 5909)	−0·2 (−12·9 to 12·4)	74 748 (63 715 to 87 463)	−5·9 (−10·0 to −2·2)	63 516 (50 730 to 78 312)	−1·6 (−13·1 to 9·9)
Uganda	2531 (2015 to 3154)	2·4 (−9·4 to 15·3)	42 050 (35 224 to 50 418)	−4·2 (−7·4 to −0·6)	33 609 (27 179 to 41 603)	−0·2 (−10·6 to 11·0)
Zambia	1222 (907 to 1609)	6·2 (−15·1 to 30·3)	20 323 (16 876 to 24 320)	−2·5 (−6·1 to 1·2)	17 098 (12 886 to 22 139)	4·7 (−15·4 to 27·2)
**Central sub-Saharan Africa**	**10 351 (8 428 to 12 697)**	**8·1 (0·2 to 17·5)**	**210 389 (176 711 to 249 382)**	**−2·5 (−4·9 to 0·0)**	**154 408 (126 081 to 187 843)**	**5·6 (−1·1 to 13·1)**
Angola	1795 (1354 to 2336)	23·7 (1·2 to 57·1)	36 872 (30 732 to 43 858)	−3·1 (−6·6 to 0·3)	27 363 (20 993 to 35 154)	15·4 (−3·8 to 40·7)
Central African Republic	557 (418 to 722)	2·9 (−13·0 to 17·9)	12 817 (10 652 to 15 123)	3·4 (−2·2 to 12·0)	8538 (6627 to 10 751)	4·2 (−10·7 to 19·6)
Congo (Brazzaville)	594 (477 to 725)	10·7 (−5·4 to 27·6)	11 239 (9653 to 12 938)	2·3 (−4·4 to 9·3)	8551 (6924 to 10 410)	7·9 (−7·4 to 23·7)
Democratic Republic of the Congo	6905 (5626 to 8637)	4·8 (−3·7 to 15·4)	141 576 (118 678 to 168 958)	−3·4 (−6·8 to 0·5)	103 557 (83 985 to 127 321)	3·3 (−4·1 to 11·8)
Equatorial Guinea	93 (60 to 131)	11·8 (−20·1 to 46·6)	1716 (1452 to 2015)	−4·4 (−8·4 to −0·8)	1267 (847 to 1769)	4·0 (−22·7 to 33·2)
Gabon	407 (326 to 504)	10·0 (−5·0 to 26·1)	6170 (5123 to 7355)	−2·0 (−5·3 to 1·8)	5132 (4121 to 6334)	6·6 (−6·7 to 21·4)

Data are n (95% UI) or percentage change in age-standardised rates (95%UI). DALYs= disability-adjusted life-years. SDI=Socio-demographic Index. UI=uncertainty interval.

The number of deaths due to dementia increased by 148% (140–157) between 1990 and 2016. Globally in 2016, dementia was the fifth-largest cause of death (2·4 million [95% UI 2·1–2·8] deaths) after ischaemic heart disease, chronic obstructive pulmonary disease, intracerebral haemorrhage, and ischaemic stroke. In 2016, deaths due to dementia accounted for 4·4% (95% UI 3·8–5·1) of total deaths but 8·6% (7·4–10·1) of deaths in individuals aged more than 70 years (2·2 million [1·9–2·6] deaths),^13^ making dementia the second largest cause of death in this age group after ischaemic heart disease. Globally, dementia caused 28·8 million (24·5–34·0) DALYs, making it the 23rd largest cause of DALYs globally in 2016, up from 41st in 1990.^16^ Dementia accounted for 1·2% (95% UI 1·0–1·4) of DALYs across all ages. Over the age of 70 years, this increased to 6·3% (5·4–7·5) of DALYs (23·9 million DALYs, 20·1–28·6).^16^

More women than men died from dementia in 2016 (1·5 million, 95% UI 1·3–1·8 *vs* 0·8 million, 0·7–1·0). The age-standardised death rates in women were also higher than in men, in line with a higher prevalence in women than in men, indicating the female predominance was not simply due to the longer lifespan of women. Age-standardised global prevalence in females was 1·17 times (1·17–1·18) the age-standardised prevalence in males in 2016, with more women globally affected by dementia (27·0 million, 95% UI 23·3–31·4) than men (16·8 million, 14·4–19·6).

**Figure 2:**
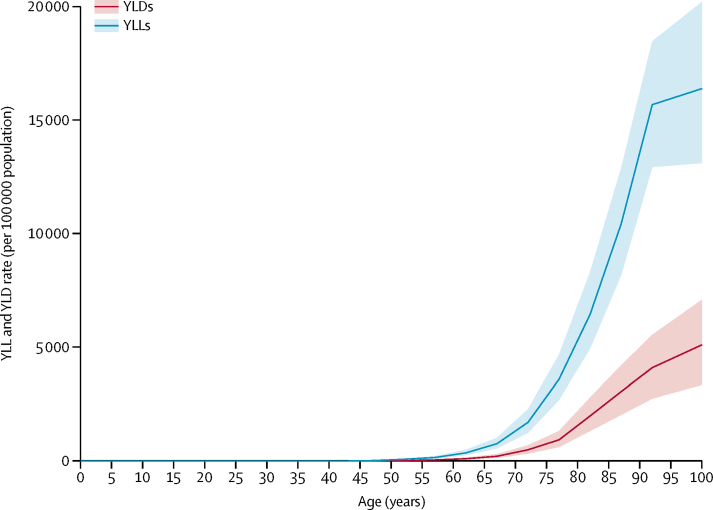
Global years lived with disability (YLDs) and years of life lost (YLLs) rates per 100 000 population due to Alzheimer's disease and other dementias by age, 2016 Values are plotted at the midpoint of 5-year age categories. Shaded areas show 95% uncertainty intervals.

**Figure 3:**
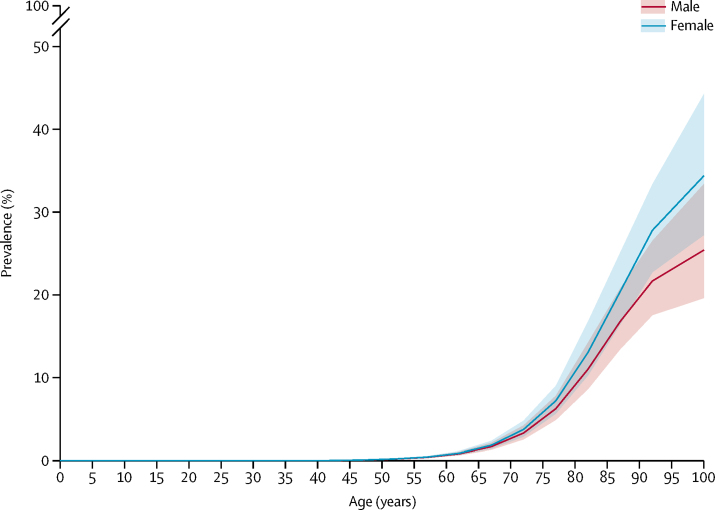
Global age-standardised prevalence of Alzheimer's disease and other dementias by sex, 2016 Prevalence expressed as the percentage of the population that was affected by the disease. Values are plotted at the midpoint of 5-year age categories. Shading indicates 95% uncertainty intervals.

Both YLLs and YLDs increased sharply with age (figure 2). However, YLL rates increased faster with age and were much higher than YLD rates at the oldest ages. Prevalence also increased substantially with age in both men and women, approximately doubling every 5 years between the ages of 50 and 80 years, after which the increase slowed owing to the high prevalence in the oldest ages (figure 3).

**Figure 4:**
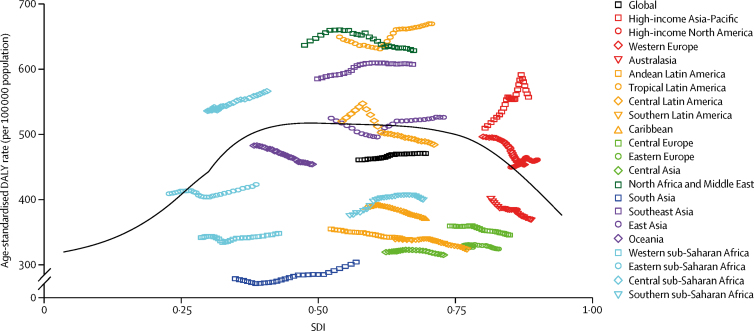
Age-standardised disability-adjusted-life-year (DALY)rates for Alzheimer's disease and other dementias by Socio-demographic Index (SDI), 1990–2016 The black line represents expected values of age-standardised DALY rates for each value of SDI.

There was no clear pattern between age-standardised DALY rates for the 21 GBD world regions and SDI over the 1990–2016 estimation period (figure 4). At each SDI level, there was a large amount of heterogeneity between DALY rates due to dementia. There was no uniformity between regions in the changes over time in DALY rates, with some regions increasing and others decreasing over the 1990–2016 period.

Overall, 6·4 million (95% UI 3·4–10·5) DALYs (22·3% [95% UI 11·8–35·1] of DALYs due to dementia) in 2016 could be attributed to risk factors analysed as part of GBD: 3·5 million (1·4–6·7) DALYs (12·2% [4·7–23·1]) were attributed to high BMI; 2·1 million (0·4–4·9) DALYs (7·4% [1·6–16·1]) to high fasting plasma glucose; 1·3 million (0·6–2·0) DALYS (4·4% [2·0–6·7]) to smoking; and 18·9 thousand (7·8–34·1) DALYs (0·07% [0·03–0·12]) to a diet high in sugar-sweetened beverages. There was no significant difference in the proportion of all dementia DALYs in 2016 explained by risk factors in men (24·7%, 12·7–37·6) compared with women (20·8%, 8·6–35·7). More detailed estimates, including values for all years from 1990–2016, and all locations, all age groups, and both sexes, can be found in our online results tool.

## Discussion

We estimated that in 2016, 27·0 million (95% UI 23·3–31·4) women and 16·8 million (14·4–19·6) men lived with dementia in the 195 countries and territories that were included in the 2016 round of GBD. The number of prevalent cases of dementia more than doubled from 1990 to 2016, contrasting with relatively minor changes in age-standardised prevalence and pointing to population ageing and growth as the main drivers of the increase. The numbers of DALYs and age-standardised DALY rates showed similar patterns to the prevalence figures. Dementia was the fifth leading cause of cause of death in 2016.

Our overall global estimate of 43·8 million people living with dementia in 2016 is close to the estimate in the World Alzheimer Report 2015 of 46·8 million for 2015.^2^ Additionally, the GBD estimate of a doubling in number of prevalent cases and a 148% (140–157) increase in dementia deaths over the 26-year period from 1990 to 2016 is of the same order as the doubling time of 20 years previously reported.^2^, ^3^ However, the previous studies reported results only at the region level and did not use data across regions to generate estimates for all countries.

The increase in the number of cases of dementia is of even more importance given that there is currently no effective disease-modifying cure or treatment for the disease.^30^ Additionally, even when clinical trials are initiated, many more end in failure than success in bringing a new disease-modifying drug to market, with ratios of more than 100:1 compared with the 14·6:1 pharmaceutical industry average.^31^, ^32^ Without potential treatments, increasing numbers of cases will pose undue burden on individuals who have dementia, their caregivers, and health-care systems more generally.

In GBD 2016, only four risk factors were judged to have sufficient evidence for a causal link to Alzheimer's disease and other dementias: high BMI, high fasting plasma glucose, smoking, and high intake of sugar-sweetened beverages.^23^ In GBD, the effect of high intake of sugar-sweetened beverages was posited to be mediated through BMI on the basis of scientific literature linking BMI with dementia, but sugar-sweetened beverages as such explained only a negligible fraction of dementia burden attributed to risks. GBD is continuously reviewing new evidence for risk-outcome pairs and will update estimates of dementia burden attributable to risks in upcoming cycles for risk factors that meet GBD criteria for causality. A *Lancet* Commission Report^5^ suggested that modifiable risk factors including hearing loss, education, smoking, depression, physical inactivity, social isolation, diabetes, and obesity could account for as much as 35% of dementia burden. In the context of our finding of a doubling of the prevalence of this terminal disease every 5 years over age 50 years, and the absence of a cure, the impetus to examine risk factors is clear.

Furthermore, the timing of interventions and prevention efforts focused on modifiable risk factors for dementia warrants further investigation. The prodrome of disease is thought to be long, with evidence pointing to a length of 20–30 years, but potentially longer.^33^ The inability to identify individuals accurately in the prodromal stage complicates the study of risk factors. Efforts to explore the timing of risk factors during this period are further limited by the span of observational studies and the short length of randomised controlled trials, as well as uncertainty over whether exposures are causative, are bystanders of highly correlated factors, or are even early symptoms of disease. Because of these issues, there are so far no official international lifestyle guidelines for preventing dementia.^34^ However, attention on the effects of dementia risks is increasing, and WHO has created a group tasked with the development of risk-reduction guidelines.^35^

By 2050, the number of people living with dementia could be around 100 million.^36^ Tackling this will require training of health professionals, as well as planning and building facilities to cater to increasing numbers individuals with dementia. The cost of care for those living with dementia is also very high, especially in high-income countries. According to recent estimates, in the USA the cost was US$818 million in 2015, an increase of 35% since 2010.^37^ Since ageing is expected to continue, the only way to reduce burden and associated costs is to identify effective preventive or treatment measures. Despite the low return on research investment in dementia in the past, the size of the burden and its increasing trend warrant a continued effort to find effective means of intervening. Until such breakthroughs are made, dementia will constitute an increasing challenge to health-care systems across the globe.

There were several limitations, separate from the broader limitations of GBD, relevant to the modelling process for dementia. First, to correct for changes in coding practices in cause of death data, we selected the countries that were most willing to code to dementia as a cause of death per prevalent case, and we assumed that certification and coding practices in these countries are correct. Although coding practices are probably not perfect in these countries, we selected them as the best available benchmark. Second, in this correction we then assumed that the excess mortality derived from these countries applied to all countries across the entire timeseries. Although this assumption was clearly approximate, it was necessary to address the changes in coding practices that led to large changes over the study period in cause of death data from countries with high-quality vital registration. Third, in correcting for the bias in cause of death data, we relied on prevalence data to determine patterns in geographical distribution of both prevalence and mortality. Although we had a large number of data sources from western Europe, east Asia, high-income Asia-Pacific, and high-income North America, for 13 of the 21 regions we had fewer than five prevalence sources. Fourth, there was a large amount of heterogeneity in the ways in which dementia was diagnosed within the available data. Of the 237 available data sources, 230 different diagnostic procedures were used.

Although most of the data ultimately classified dementia cases using the DSM or ICD definitions, differences between different versions of the DSM criteria could have led to differences in prevalence estimates, as suggested in a meta-regression analysis of prevalence studies in China.^38^ Even if the same screening test was used, different studies often used different cutoff scores. For example, although 42% of studies used the Mini-Mental State Examination in the screening phase, the absolute cutoff scores that were used ranged from 18 to 28, and other studies used different cutoff scores by educational attainment level. In this round of GBD we did not find a way to correct for such bias because of the extreme variability in methods, so it is likely that part of the observed variation in prevalence was due to measurement bias rather than reflecting true geographical variation.

A potential next step in the GBD is to consider dividing dementia into subtypes, as these might have different epidemiological features and potentially different prevention and treatment strategies. A first subdivision could be Alzheimer's disease dementia, vascular dementia, and remaining types. The challenges of subdividing include sparse data and the complication of how to handle mixed types of dementia.^39^ However, increased use of biomarkers in the classification of dementia and Alzheimer's disease might help facilitate subdivision.^40^, ^41^ Additionally, the data on severity distributions over age rely on few data sources and can be strengthened. We also aim to expand our data coverage through increased use of claims data and other data types, including general practitioner data, which have been used to estimate dementia prevalence.^42^, ^43^, ^44^

Monitoring trends in dementia is difficult because of the extreme variation in cause of death coding practices and the large heterogeneity in case-ascertainment methods. Although previous guidelines have been developed to systematise the reporting of neurological disorders generally, because of the diagnostic challenges noted with dementia, disease-specific guidelines are warranted and resources should be directed towards creating and implementing more systematic methods.^45^ The GBD study will continue to update its estimates for dementia annually, and estimates might become more robust if data collection methods improve. Additionally, as new data become available on risk factors for dementia that meet GBD criteria for causal links, they can be incorporated into future iterations of GBD.
