# Supplementary material for: Global, regional, and national burden of Alzheimer's disease and other dementias, 1990–2016: a systematic analysis for the Global Burden of Disease Study 2016
Source: Lancet Neurol. 2019 Jan;18(1):88–106. doi: 10.1016/S1474-4422(18)30403-4 (PMC6291454; doi:10.1016/S1474-4422(18)30403-4)
Supplement: Supplementary appendix [file mmc1.pdf]

THE LANCET
Neurology

Supplementary appendix

This appendix formed part of the original submission and has been peer reviewed. We post it as supplied by the authors.

Supplement to: GBD 2016 Dementia Collaborators. Global, regional, and national burden of Alzheimer's disease and other dementias, 1990–2016: a systematic analysis for the Global Burden of Disease Study 2016. *Lancet Neurol* 2018; published online Nov 26. http://dx.doi.org/10.1016/S1474-4422(18)30403-4.

Appendix: Global, regional, and national burden of Alzheimer's disease and other dementias, 1990–2016: a systematic analysis for the Global Burden of Disease Study 2016

Appendix 1: Summary Methods for the Global Burden of Disease: Neurological Disorders

The Institute for Health Metrics and Evaluation, with a growing collaboration of scientists, produces annual updates of the Global Burden of Disease study. Estimates span the period from 1990 to the most recent completed year. By the time of the release of GBD 2016 in September 2017, there were over 2,700 collaborators in 132 countries who contributed to this global public good. Annual updates allow incorporation of new data and method improvements to ensure that the most up-to-date information is available to policy makers in a timely fashion to help make resource allocation decisions. In this analysis, we have aggregated results from GBD 2016 for 15 disease and injury outcomes that are generally cared for by neurological services. These include infectious conditions (tetanus, meningitis, encephalitis), stroke, brain and other nervous system cancers, traumatic brain injury, and spinal cord lesions which are classified outside the more narrowly defined category of neurological disorders in GBD (ie, Alzheimer's disease and other dementias, Parkinson's disease, multiple sclerosis, motor neuron disease, idiopathic epilepsy, migraine, tension-type headache, and a rest category of less common other neurological disorders). Compared to a previous analysis based on GBD 2015,[1] we were able to add the non-fatal outcomes of traumatic brain injury and spinal cord lesion, and medication overuse headache is no longer included as a separate cause but is quantified as a consequence of the underlying headache types.

In the methods section of this overview paper we present a summary of the general methods of the global burden of disease. In the accompanying disease-specific papers we concentrate on methods that are specific to each disorder. The guiding principle of GBD is to assess health loss due to mortality and disability comprehensively, where we define disability as any departure from full health. In GBD 2016, estimates were made for 195 countries and territories, and 579 subnational locations, for 27 years starting from 1990, for 23 age groups and both sexes. Deaths were estimated for 264 diseases and injuries, while prevalence and incidence were estimated for 328 diseases and injuries. In order to allow meaningful comparisons between deaths and non-fatal disease outcomes as well as between diseases, the data on deaths and prevalence are summarised in a single indicator, the disability-adjusted life-year (DALY). DALYs are the sum of years of life lost (YLLs) and years lived with disability (YLDs). YLLs are estimated as the multiplication of counts of death and a standard, "ideal", remaining life expectancy at the age of death. The standard life expectancy is derived from the lowest observed mortality rates in any population in the world greater than 5 million.[2] YLDs are estimated as the product of prevalence of individual consequences of disease (or "sequelae") times a disability weight that quantifies the relative severity of a sequela as a number between zero (representing "full health") and 1 (representing death). Disability weights have been estimated in nine population surveys and an open-access internet survey in which respondents are asked to choose the "healthier"[3] between random pairs of health states that are presented with a short description of the main features.

All-cause mortality rates are estimated from vital registration data in countries with complete coverage. For other countries, the probabilities of death before age 5 and between ages 15 and 60 are estimated from censuses and surveys asking mothers to provide a history of children ever born and those still alive, and surveys asking adults about siblings who are alive or deceased. Using model life tables, these probabilities of death are transformed into age-specific death rates by location, year, and sex. GBD has collated a large database of cause of death data from vital registrations and verbal autopsy surveys in which relatives are asked a standard set of questions to ascertain the likely cause of death, supplemented with police and mortuary data for injury deaths in countries with no other data. For

countries with vital registration data, the completeness is assessed with demographic methods based on comparing recorded deaths with population counts between two successive censuses. The cause of death information is provided in a large number of different classification systems based on versions of the International Classification of Diseases or bespoke classifications in some countries. All data are mapped into the disease and injury categories of GBD. All classification systems contain codes that are less informative because they lack a specific diagnosis (eg, unspecified cancer) or refer to codes that cannot be underlying cause of death (eg, low back pain or senility) or are intermediate causes (eg, heart failure or sepsis). Such deaths are redistributed to more precise underlying causes of death.[4] After these redistributions and corrections for under-registration, the data are analysed in CODEm (cause of death ensemble model), a highly systematised tool that runs many different models on the same data and chooses an ensemble of models that best reflects all the available input data. Models are chosen with variations in the statistical approach ("mixed effects" of spatiotemporal Gaussian process regression), in the unit of analysis (rates or cause fractions), and the choice of predictive covariates. The statistical performance of all models is tested by holding out 30% of the data and checking how well a model covers the data that were held out. To enforce consistency from CODEm, the sum of all cause-specific mortality rates is scaled to that of the all-cause mortality rates in each age, sex, location, and year category.

Non-fatal estimates are based on systematic reviews of published papers and unpublished documents, survey microdata, administrative records of health encounters, registries, and disease surveillance systems. Our Global Health Data Exchange (GHDx, http://ghdx.healthdata.org/) is the largest repository of health data globally. We first set a reference case definition and/or study method that best quantifies each disease or injury or consequence thereof. If there is evidence of a systematic bias in data that used different case definitions or methods compared to reference data, we adjust those data points to reflect what its value would have been if measured as the reference. This is a necessary step if one wants to use all data pertaining to a particular quantity of interest rather than choosing a small subset of data of the highest quality only. DisMod-MR 2.1, a Bayesian meta-regression tool, is our main method of analysing non-fatal data. It is designed as a geographical cascade where a first model is run on all the world's data, which produces an initial global fit and estimates coefficients for predictor variables and the adjustments for alternative study characteristics. The global fit, adjusted by the values of random effects for each of seven GBD super-regions, the coefficients on sex and country predictors, are passed down as data to a model for each super-region together with the input data for that geography. The same steps are repeated going from super-region to 21 region fits and then to 195 fits by country and, where applicable, a further level down to subnational units. Below the global fit, all models are run separately by sex and for six time periods: 1990, 1995, 2000, 2005, 2010, and 2016. During each fit all data on prevalence, incidence, remission (ie, cure rate) and mortality are forced to be internally consistent. For most diseases, the bulk of data on prevalence or incidence are at the disease level, with fewer studies providing data on the proportions of cases of disease in each of the sequelae defined for the disease. The proportions in each sequela are pooled using DisMod-MR 2.1 or meta-analysis, or derived from analyses of patient-level datasets. The multiplication of prevalent cases for each disease sequela and the appropriate disability weight produces YLD estimates that do not yet take into account comorbidity. To correct for comorbidity, these data are used in a simulation to create hypothetical individuals in each age, sex, location, and year combination who experience no, one, or multiple sequelae simultaneously. We assume that disability weights are multiplicative rather than additive as this avoids assigning a combined disability weight value in any individual to exceed 1, ie, be worse than a

"year lost due to death". This comorbidity adjustment leads to an average scaling down of disease-specific YLDs ranging from about 2% in young children up to 17% in oldest ages.

All our estimates of causes of death are categorical: each death is assigned to a single underlying cause. This has the attractive property that all estimates add to 100%. For risks, we use a different, "counterfactual" approach, ie, answering the question: "what would the burden have been if the population had been exposed to a theoretical minimum level of exposure to a risk?" Thus, we need to define what level of exposure to a risk factor leads to the lowest amount of disease. We then analyse data on the prevalence of exposure to a risk and derive relative risks for any risk-outcome pair for which we find sufficient evidence of a causal relationship. Prevalence of exposure is estimated in DisMod-MR 2.1, using spatiotemporal Gaussian process regression, or from satellite imagery in the case of ambient air pollution. Relative risk data are pooled using meta-analysis of cohort, case-control, and/or intervention studies. For each risk and outcome pair, we evaluate the evidence and judge if the evidence falls into the categories of "convincing" or "probable" as defined by the World Cancer Research Fund.[5] From the prevalence and relative risk results, population attributable fractions are estimated relative to the theoretical minimum risk exposure level (TMREL). When we aggregate estimates for clusters of risks, eg, metabolic or behavioural risks, we use a multiplicative function rather than simple addition and take into account how much of each risk is mediated through another risk. For instance, some of the risk of high body-mass index is directly onto stroke as an outcome, but much of its impact is mediated through high blood pressure, high cholesterol, or high fasting plasma glucose, and we would not want to double count the mediated effects when we estimate aggregates across risk factors.[6]

Uncertainty is propagated throughout all these calculations by creating 1,000 values for each prevalence, death, YLL, YLD, or DALY estimate and performing aggregations across causes and locations at the level of each of the 1,000 values for all intermediate steps in the calculation. The lower and upper bounds of the 95% uncertainty interval are the 25th and 975th values of the ordered 1,000 values. For all age-standardised rates, GBD uses a standard population calculated as the non-weighted average across all countries of the percentage of the population in each five-year age group for the years 2010 to 2035 from the United Nations Population Division's World Population Prospects (2012 revision).[7,8]

GBD uses a composite indicator of sociodemographic development, the Socio-demographic Index (SDI), which reflects the geometric mean of normalised values of a location's income per capita, the average years of schooling in the population 15 and over, and the total fertility rate. Countries and territories are grouped into five quintiles of high, high-middle, middle, low-middle, and low SDI based on their 2016 values.[2]

1 GBD 2015 Neurological Disorders Collaborator Group. Global, regional, and national burden of neurological disorders during 1990-2015: a systematic analysis for the Global Burden of Disease Study 2015. *Lancet Neurol* 2017; **16**: 877–97.

2 GBD 2016 Mortality Collaborators. Global, regional, and national under-5 mortality, adult mortality, age-specific mortality, and life expectancy, 1970-2016: a systematic analysis for the Global Burden of Disease Study 2016. *Lancet Lond Engl* 2017; **390**: 1084–150.

3 Salomon JA, Haagsma JA, Davis A, *et al.* Disability weights for the Global Burden of Disease 2013 study. *Lancet Glob Health* 2015; **3**: e712-723.

4 GBD 2016 Causes of Death Collaborators. Global, regional, and national age-sex specific mortality for 264 causes of death, 1980-2016: a systematic analysis for the Global Burden of Disease Study 2016. *Lancet Lond Engl* 2017; **390**: 1151–210.

5 American Institute for Cancer Research. Food, nutrition, physical activity, and the prevention of cancer: a global perspective. Washington, DC: American Institute for Cancer Research, 2007.

6 GBD 2016 Risk Factors Collaborators. Global, regional, and national comparative risk assessment of 84 behavioural, environmental and occupational, and metabolic risks or clusters of risks, 1990-2016: a systematic analysis for the Global Burden of Disease Study 2016. *Lancet Lond Engl* 2017; **390**: 1345–422.

7 GBD 2013 Mortality and Causes of Death Collaborators. Global, regional, and national age-sex specific all-cause and cause-specific mortality for 240 causes of death, 1990-2013: a systematic analysis for the Global Burden of Disease Study 2013. *Lancet Lond Engl* 2015; **385**: 117–71.

8 United Nations Department of Economics and Social Affairs Population Division. World Population Prospects: The 2012 Revision. http://esa.un.org/unpd/wpp/Documentation/publications.htm (accessed Nov 4, 2014).

Appendix 2: Count of data sources used in non-fatal modelling for Alzheimer's disease and other dementias by 21 GBD regions in 2016

Region name	Incidence	Prevalence	Mortality	Claims data
East Asia	8	34	4	0
Southeast Asia	0	6	0	0
Oceania	0	0	0	0
Central Asia	0	0	0	0
Central Europe	0	1	0	0
Eastern Europe	0	0	0	0
High-income Asia Pacific	3	23	1	0
Australasia	2	3	0	0
Western Europe	33	63	9	0
Southern Latin America	0	1	0	0
High-income North America	10	7	1	3
Caribbean	1	3	1	0
Andean Latin America	1	1	1	0
Central Latin America	3	3	1	0
Tropical Latin America	1	5	0	0
North Africa and Middle East	0	6	0	0
South Asia	0	12	1	0
Central sub-Saharan Africa	0	3	0	0
Eastern sub-Saharan Africa	0	1	0	0
Southern sub-Saharan Africa	0	0	0	0
Western sub-Saharan Africa	2	4	1	0
**Total**	**64**	**176**	**20**	**3**

* Mortality refers to data on mortality in scientific literature, including data on standardised mortality ratio and relative risk

Appendix 3: Cause of death methods write-up[1]

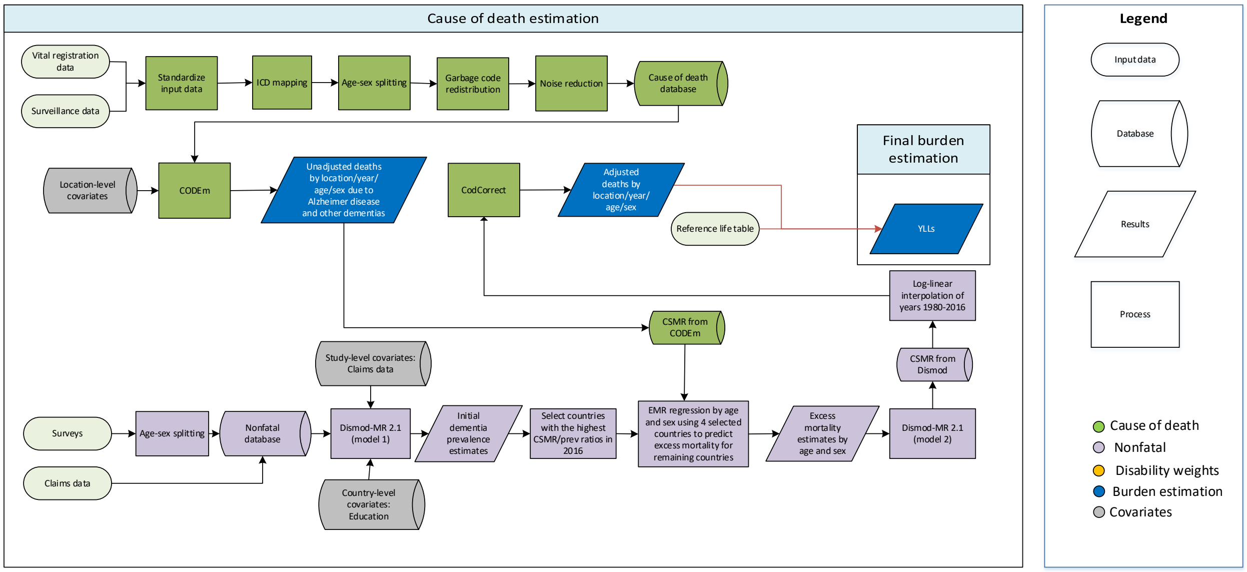

Input data

In GBD 2016, data used to estimate deaths due to Alzheimer's disease and other dementias (dementias hereafter) included mortality data from vital registration systems and prevalence data from surveys and medical claims sources.

An updated systematic review was conducted from January 2015 to October 2016, and search terms[2] were set to capture studies for all dementia, including its subtypes. The search yielded 1,208 initial hits, and 27 were marked for extraction. Inclusion criteria allowed studies that reported prevalence, incidence, remission rate, excess mortality rate, relative risk of mortality, standardised mortality ratio, or with-condition mortality rate. Studies with non-representative samples or no clearly defined sample were excluded. A flow chart documenting this review is displayed below.

[1] This section of the appendix was first published in the appendix to: GBD 2016 Causes of Death Collaborators. Global, regional, and national age-sex specific mortality for 264 causes of death, 1980–2016: a systematic analysis for the Global Burden of Disease Study 2016. The Lancet. 14 Sept 2017: 390;1151–210.

[2] ((dementia[Title/Abstract]) AND ((incidence[Title/Abstract]) or (prevalence[Title/Abstract])) AND ("2015/01/23"[Date - Publication] : "3000"[Date - Publication])

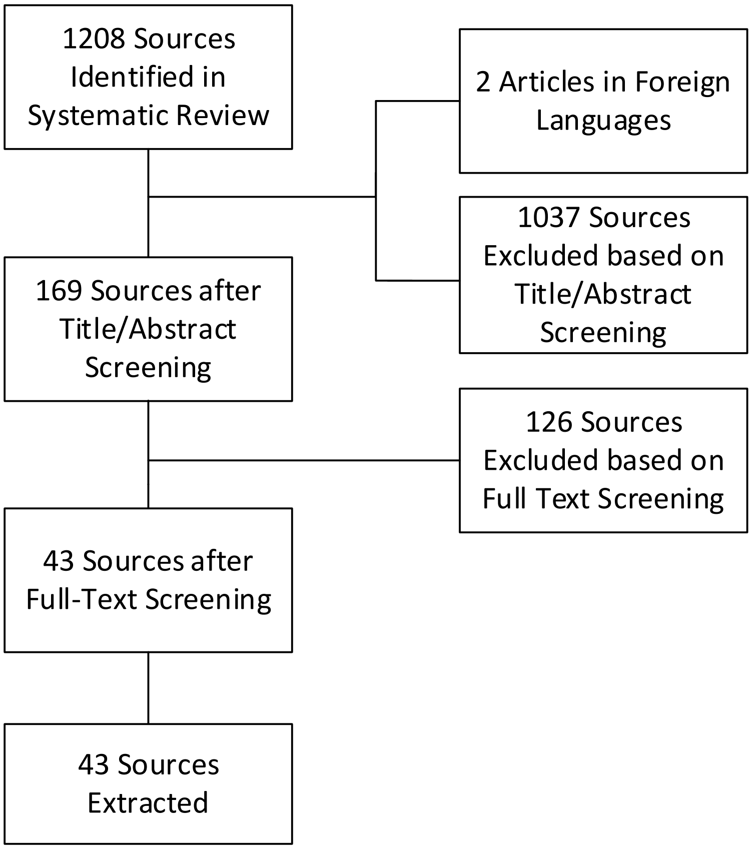

Modelling strategy

Overview

Dementia mortality rates have increased more than five-fold since 1980 in high-quality vital registration systems such as in the USA and Scandinavia. We have not seen an equivalent increase in prevalence and incidence data sources. If at all, there has been a modest decline in incidence and prevalence of dementia in studies in the UK and the USA.[3] [4] Also, the greater than 20-fold variation in mortality rates of dementia between countries is much greater than the four-fold difference in prevalence and incidence between countries. As it is unlikely that case fatality from dementia has dramatically increased over the time period and that it would differ by a very large margin between countries, the hypothesis is that certifying and coding practices have changed over time and at a different pace between countries. To avoid spurious large trends over time in the fatal component of the burden of dementia, we decided for GBD 2013 to make dementia mortality rates consistent with the most recent rates relative to prevalence in countries that are most likely to certify or code dementia as an underlying cause of death. This approach was applied again for GBD 2016, described further below.

[3] Akushevich I, Kravchenko J, Ukraintseva S, Arbeev K, Yashin AI. Time trends of incidence of age-associated diseases in the US elderly population: Medicare-based analysis. *Age and ageing*. 2013 Jul 1;42(4):494-500.

[4] Matthews FE, Arthur A, Barnes LE, Bond J, Jagger C, Robinson L, Brayne C, Medical Research Council Cognitive Function and Ageing Collaboration. A two-decade comparison of prevalence of dementia in individuals aged 65 years and older from three geographical areas of England: results of the Cognitive Function and Ageing Study I and II. *The Lancet*. 2013 Nov 1;382(9902):1405-12.

Modelling steps

First, we ran a CODEm model for dementia and extracted the mortality rates by age, sex, and geography for 2016. The covariates used in this model are displayed below.

Level	Covariate	Direction
1	Diabetes age-specific proportion	+
	Mean BMI	+
	Cholesterol (total, mean per capita)	+
	Systolic blood pressure (mmHg)	+
2	Animal fats (kcal per capita)	+
	Latitude over 45 (proportion)	+
	Red meat consumption, adjusted (g)	+
	Healthcare Access and Quality index	-
3	Education (years per capita)	-
	LDI per capita (I$ per capita)	0
	Sanitation (proportion with access)	-
	Improved water source (proportion with access)	-

Second, we ran a DisMod-MR 2.1 model with all data on incidence, prevalence, and mortality risk (RR, SMR, or with-condition mortality rates) and a setting of zero remission and extracted 2016 prevalence by age, sex, and geography. To account for potential systematic differences between USA medical claims and survey data, we crosswalked for each year of claims data.

Third, we selected countries where the sum of cause-specific mortality rate to prevalence ratio for males and females exceeded 0.4 (excluding small island nations and those without vital registration). This resulted in choosing the United States, Sweden, Finland, and Puerto Rico. The choice to pick fewer countries for this regression compared to GBD 2015, which used 30 countries in the EMR regression, was motivated by a desire to reduce the spread in EMR values, as countries used in the regression retain their original EMR values.

Fourth, we used a linear effects regression with dummies on age group and sex to predict excess mortality (ie, the ratio of cause-specific mortality rate and prevalence) by age and sex, the results of which are found in the tables below.

Table: Fixed effect coefficients of EMR regression. Outcome: ln(EMR)

Independent variables	Coef	Std. error	P value	95% Confidence interval	
Male	0.047	0.054	0.394	-0.06	0.153
Age 40-59	-3.299	0.115	0.000	-3.524	-3.073
Age 60-64	-2.627	0.115	0.000	-2.852	-2.401
Age 65-69	-2.395	0.115	0.000	-2.62	-2.169
Age 70-74	-2.068	0.115	0.000	-2.293	-1.842
Age 75- 79	-1.686	0.115	0.000	-1.911	-1.461
Age 80-84	-1.362	0.115	0.000	-1.587	-1.137
Age 85-89	-0.949	0.115	0.000	-1.175	-0.724
Age 90-94	-0.426	0.115	0.001	-0.651	-0.2

Constant	-1.539	0.086	0.000	-1.707	-1.371

Table: Predicted EMR values by age and sex (95% CI)

	Male	Female
Age 40-59	0.008 (0.007 - 0.01)	0.008 (0.007 - 0.009)
Age 60-64	0.016 (0.014 - 0.019)	0.016 (0.013 - 0.018)
Age 65-69	0.021 (0.017 - 0.024)	0.02 (0.017 - 0.023)
Age 70-74	0.029 (0.024 - 0.034)	0.027 (0.023 - 0.032)
Age 75- 80	0.042 (0.035 - 0.049)	0.04 (0.034 - 0.047)
Age 80-84	0.058 (0.049 - 0.068)	0.055 (0.047 - 0.064)
Age 85-89	0.088 (0.074 - 0.104)	0.084 (0.07 - 0.098)
Age 90-94	0.147 (0.124 - 0.174)	0.14 (0.118 - 0.165)
Age 95+	0.226 (0.19 - 0.268)	0.216 (0.183 - 0.254)

Fifth, these estimates were added to a second DisMod-MR 2.1 model as pertaining to the full 1990–2016 estimation period. For the four countries included in the regression, we retained their age- and sex-specific ratios and entered those also as pertaining to the full 1990–2016 estimation period. Age-standardised years of education was used as a country-level covariate. We excluded data for standardised mortality ratio, with-condition mortality rate, and relative risk as we wanted to estimate cause-specific mortality rates that were consistent with the level of excess mortality from the four chosen countries in 2016.

Sixth, we took the predictions of cause-specific mortality by age, sex, geography, and year that DisMod-MR 2.1 calculated as being consistent with the data on incidence, prevalence, and the priors on excess mortality from step five.

Seventh, because DisMod-MR 2.1 produces estimates in five-year intervals only, we expanded the time series by log-linear interpolation. Values for 1980–1990 were generated using a regression on the entire time series with Socio-demographic Index included as a predictor.

Lastly, before adding the dementia mortality estimates into CodCorrect, we proportionately retrieved the difference in deaths between those estimated in CODEm and those estimated in step 7 from a set of "target causes" which were identified as causes of death in cohort studies of persons with dementia. The target causes included lower respiratory infections, protein-energy malnutrition, other nutritional deficiencies, cerebrovascular disease, interstitial nephritis and urinary tract infections, decubitus ulcer, and pulmonary aspiration and foreign body in airway.[5,6,7,8] More information on this process is located in section 4 of the appendix.

[5] Brunnström HR, Englund EM. Cause of death in patients with dementia disorders. *European journal of neurology*. 2009 Apr 1;16(4):488-92.
[6] Thomas BM, Starr JM, Whalley LJ. Death certification in treated cases of presenile Alzheimer's disease and vascular dementia in Scotland. *Age and Ageing.* 1997 Sep 1;26(5):401-6.
[7] Todd S, Barr S, Roberts M, Passmore AP. Survival in dementia and predictors of mortality: a review. *International journal of geriatric psychiatry*. 2013 Nov 1;28(11):1109-24.
[8] Keene J, Hope T, Fairburn CG, Jacoby R. Death and dementia. *International journal of geriatric psychiatry.* 2001 Oct 1;16(10):969-74.

Appendix 4: Non-fatal methods write-up[9]

Alzheimer Disease and Other Dementias

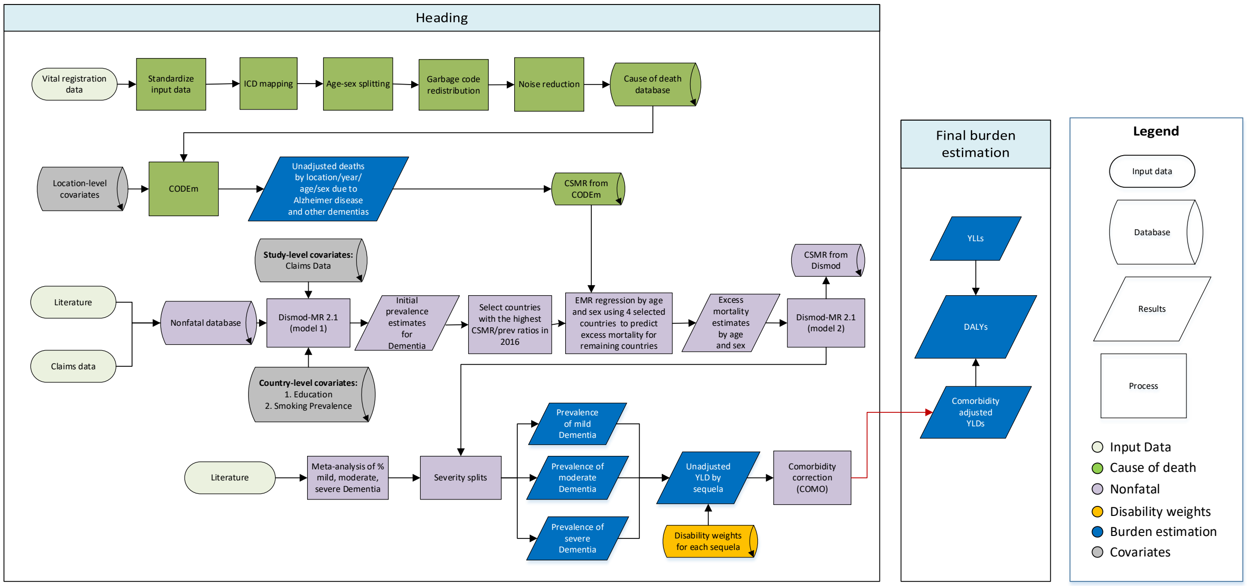

Input data and methodological summary

Case definition

Dementia is a progressive, degenerative, and chronic neurological disorder typified by memory impairment and other neurological dysfunctions. For the purposes of GBD 2016, we use the Diagnostic and Statistical Manual of Mental Disorders III, IV, or V, or ICD case definitions as the reference. A wide array of diagnostic and screening instruments exists, including Clinical Dementia Rating scale (CDR), Mini Mental State Examination (MMSE), and the Geriatric Mental State (GMS). For severity rating purposes we use the CDR as the reference. The relevant ICD-10 codes for dementia are F00, F01, F02, F03, G30, and G31. The ICD-9 codes are 290, 291.2, 291.8, 294, and 331.

Unlike most causes in the Global Burden of Disease project, dementia mortality and morbidity estimates are modelled jointly. This is because of marked discrepancies between prevalence data and cause of death data. Specifically, prevalence data suggest little to no variation over time (eg, 1990–2016) whereas age-standardised mortality rates in vital registrations in high-income countries have increased multiple times over this same period. Additionally, prevalence variation between countries is much smaller than the variation in death rates assigned to dementia in vital registration. We attribute these discrepancies to changing coding practices rather than epidemiological change.

[9] This section of the appendix was first published in the appendix to: GBD 2016 Disease and Injury Incidence and Prevalence Collaborators. Global, regional, and national incidence, prevalence, and years lived with disability for 328 diseases and injuries for 195 countries, 1990–2016: a systematic analysis for the Global Burden of Disease Study 2016. The Lancet. 14 Sept 2017: 390; 1211–59.

Because of this joint procedure, descriptions of the mortality estimation process are included where relevant.

Input data

Model inputs

To inform our estimates of burden due to dementia, we use mortality data from vital registration systems, as well as prevalence data from surveys and administrative data such as claims sources.

An updated systematic review was conducted covering January 2015 to October 2016, and search terms[10] were set to capture studies for all dementia, including its subtypes. The search yielded 1,208 initial hits and 27 were marked for extraction. Inclusion criteria allowed studies that reported prevalence, incidence, remission rate, excess mortality rate, relative risk of mortality, standardised mortality ratio, or with-condition mortality rate. Studies with no clearly defined sample were excluded. A flow chart documenting this review is displayed below.

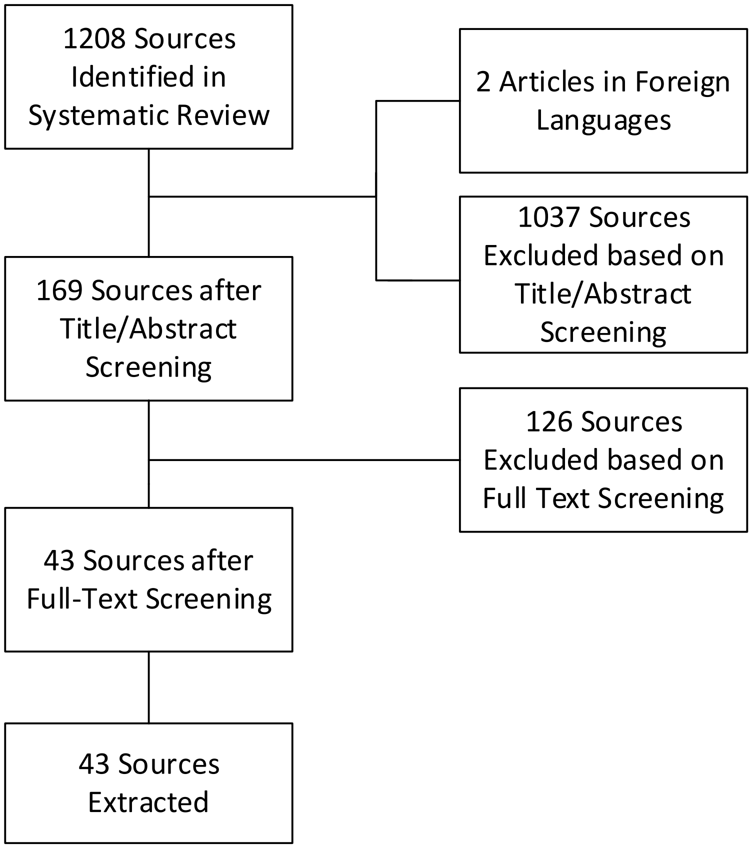

[10] ((dementia[Title/Abstract]) AND ((incidence[Title/Abstract]) or (prevalence[Title/Abstract])) AND ("2015/01/23"[Date - Publication] : "3000"[Date - Publication])

Additionally, a table describing the density and distribution of the epidemiological data available for GBD 2016 is presented below:

	Prevalence	Incidence	Mortality risk	Severity
Studies	174	60	15	17
GBD world regions	17	10	9	8

Severity splits

In GBD 2013 (and still used in GBD 2016), we extracted data from studies reporting on mild, moderate, and severe dementia. As the data indicate an age pattern with greater proportions with more severe disease in the very old, we restricted our analyses to studies reporting on severity <70, 70-79, and 80+ ages. Most of these studies reported severity based on the CDR scale: CDR=1 as mild, CDR=2 as moderate, and CDR=3 as severe dementia. Other studies report staging of dementia according to MMSE; the Functional capacity scale; the Cambridge Mental Disorders of the Elderly Examination (CAMDEX); the scale of Hughes; and the Geriatric Mental State (GMS). This year we excluded all studies that used the DSM III criteria, as we found that these sources reported systematically higher severities. We used a random effects meta-analysis to pool the data by severity level.

We multiplied estimations of prevalence (country-year-sex-age-specific) by the fractions of mild, moderate, and severe dementia and estimated 95% uncertainty intervals at the 1,000-draw level.

Severity level	Lay description	DW (95% CI)	Severity distribution
Mild	The person has some trouble remembering recent events and finds it hard to concentrate and make decisions and plans.	0.069 (0.046–0.099)	<70: 79% (65–86%)<br>70-79: 71% (51–84%)<br>80+: 61% (44–76%)
Moderate	The person has memory problems and confusion, feels disoriented, at times hears voices that are not real, and needs help with some daily activities.	0.377 (0.252–0.508)	<70: 17% (5–24%)<br>70-79: 19% (8–37%)<br>80+: 26% (16–34%)
Severe	The person has complete memory loss, no longer recognises close family members, and requires help with all daily activities.	0.449 (0.304–0.595)	<70: 4% (2–42%)<br>70-79: 9% (8–17%)<br>80+: 12% (9–24%)

Modelling strategy

As mentioned above, the estimation of morbidity due to dementia occurs in conjunction with the mortality estimation.

First, we ran a CODEm model for dementia and extracted the mortality rates by age, sex, and geography for 2016.

Second, we ran a DisMod-MR 2.1 model with all data on incidence, prevalence, and mortality risk (relative risk, standardised mortality ratio, or with-condition mortality rates) and a setting of zero

remission and extracted 2016 prevalence by age, sex, and geography. To account for potential systematic differences between claims and survey data, we crosswalked for each year of claims data.

Third, we selected countries where the sum of cause-specific mortality rate to prevalence ratio for males and females exceeded 0.4 (excluding small island nations and those without vital registration). This resulted in choosing the United States, Sweden, Finland, and Puerto Rico. The choice to pick fewer countries for this regression compared to GBD 2015, which used 30 countries in the EMR regression, was motivated by a desire to reduce the spread in EMR values, as countries used in the regression retain their original EMR values.

Fourth, we used a linear effects regression with dummies on age group and sex to predict excess mortality (ie, the ratio of cause-specific mortality rate and prevalence) by age and sex, the results of which are found in the tables below.

Table: Fixed effect coefficients of EMR regression. Outcome: ln(EMR)

Independent variables	Coef	Std. error	P value	95% Confidence interval	
Male	0.047	0.054	0.394	-0.06	0.153
Age 40-59	-3.299	0.115	0.000	-3.524	-3.073
Age 60-64	-2.627	0.115	0.000	-2.852	-2.401
Age 65-69	-2.395	0.115	0.000	-2.62	-2.169
Age 70-74	-2.068	0.115	0.000	-2.293	-1.842
Age 75- 79	-1.686	0.115	0.000	-1.911	-1.461
Age 80-84	-1.362	0.115	0.000	-1.587	-1.137
Age 85-89	-0.949	0.115	0.000	-1.175	-0.724
Age 90-94	-0.426	0.115	0.001	-0.651	-0.2
Constant	-1.539	0.086	0.000	-1.707	-1.371

Table: Predicted EMR values by age and sex (95% CI)

	Male	Female
Age 40-59	0.008 (0.007 - 0.01)	0.008 (0.007 - 0.009)
Age 60-64	0.016 (0.014 - 0.019)	0.016 (0.013 - 0.018)
Age 65-69	0.021 (0.017 - 0.024)	0.02 (0.017 - 0.023)
Age 70-74	0.029 (0.024 - 0.034)	0.027 (0.023 - 0.032)
Age 75- 80	0.042 (0.035 - 0.049)	0.04 (0.034 - 0.047)
Age 80-84	0.058 (0.049 - 0.068)	0.055 (0.047 - 0.064)
Age 85-89	0.088 (0.074 - 0.104)	0.084 (0.07 - 0.098)
Age 90-94	0.147 (0.124 - 0.174)	0.14 (0.118 - 0.165)
Age 95+	0.226 (0.19 - 0.268)	0.216 (0.183 - 0.254)

Fifth, these estimates were added to a second DisMod-MR 2.1 model as pertaining to the full 1990–2016 estimation period. For the four countries included in the regression, we retained their age- and sex-specific ratios and entered those also as pertaining to the full 1990–2016 estimation period. Thus, the model reflects the cause-specific mortality rate if all countries over time would have had the average propensity to code to dementia as an underlying cause of death similar to the selected four countries in 2016.

In this model, we assumed zero remission as well as zero excess mortality and incidence until age 40. Because of lack of consistency between prevalence and incidence data, we excluded incidence data from the final model. In a few locations we found good consistency between prevalence and incidence, and these were locations where incidence and prevalence were collected as part of the same study. In other locations (Beijing, Australia, Italy, Canada, various states in the USA, Mexico, and Nigeria) we noted that DisMod-MR 2.1 was pushing the fit above the available prevalence data and below incidence – "averaging the difference." In all cases the incidence and prevalence data were collected by different studies. We decided to drop the incidence estimates as measuring incidence of dementia when symptoms are still mild is more prone to measurement bias than measuring prevalence when the diagnosis has become more obvious over time.

The table below provides additional information on the country covariates used in this model, as well as beta and exponentiated beta values.

Variable	Measure	Beta	Exponentiated Beta Value (CI)
Mean years of education, age-standardised	prevalence	-0.053	0.95 (0.85–1.00)
Smoking prevalence, age-standardised, both sexes	prevalence	0.11	1.11 (1.06–1.17)
US claims data 2000	prevalence	-0.69	0.50 (0.48–0 .55)
US claims data 2010	prevalence	-0.25	0.78 (0.75–0.86)
US claims data 2012	prevalence	-0.25	0.78 (0.75–0.86)

As described above, we used crosswalks to standardise the claims data relative to existing literature data. Age-standardised education was used as a proxy for general brain health/use that may be protective of dementia – specifically Alzheimer's disease. Smoking prevalence (age-standardised, both sexes) was also used as a covariate to guide estimates, as the literature has shown a positive relationship between smoking and dementia.

Appendix 5: Sources used for the severity analysis

Andersen K, Lolk A, Nielsen H, Andersen J, Olsen C, Kragh-Sørensen P. Prevalence of very mild to severe dementia in Denmark. Acta Neurol Scand. 1997; 96(2): 82-7.

D'Alessandro R, Gallassi R, Benassi G, Morreale A, Lugaresi E. Dementia in subjects over 65 years of age in the Republic of San Marino. Br J Psychiatry. 1988; 182-6.

Heeren TJ, Lagaay AM, Hijmans W, Rooymans HG. Prevalence of dementia in the "oldest old" of a Dutch community. J Am Geriatr Soc. 1991; 39(8): 755-9.

Juva K, Sulkava R, Erkinjuntti T, Valvanne J, Tilvis R. Prevalence of dementia in the city of Helsinki. Acta Neurol Scand. 1993; 87(2): 106-10.

Meller I, Fichter M, Schröppel H, Beck-Eichinger M. Mental and somatic health and need for care in octo- and nonagenerians. An epidemiological community study. Eur Arch Psychiatry Clin Neurosci. 1993; 242(5): 286-92.

O'Connor DW, Pollitt PA, Hyde JB, Fellows JL, Miller ND, Brook CP, Reiss BB, Roth M. The prevalence of dementia as measured by the Cambridge Mental Disorders of the Elderly Examination. Acta Psychiatr Scand. 1989; 79(2): 190-8.

Pfeffer RI, Afifi AA, Chance JM. Prevalence of Alzheimer's disease in a retirement community. Am J Epidemiol. 1987; 125(3): 420-36.

Appendix 6: GATHER checklist of information that should be included in reports of global health estimates, with description of compliance and location of information for GBD 2016

#	GATHER checklist item	Description of compliance	Reference
**Objectives and funding**			
1	Define the indicators, populations, and time periods for which estimates were made.	Narrative provided in paper and appendix describing indicators, definitions, and populations	Main text (Methods) and appendix
2	List the funding sources for the work.	Funding sources listed in paper	Summary (Funding)
**Data Inputs**			
For all data inputs from multiple sources that are synthesised as part of the study:			
3	Describe how the data were identified and how the data were accessed.	Narrative description of data seeking methods provided	Main text (Methods) and appendix
4	Specify the inclusion and exclusion criteria. Identify all ad-hoc exclusions.	Narrative about inclusion and exclusion criteria by data type provided; ad hoc exclusions in cause-specific write-ups	Main text (Methods) and appendix
5	Provide information on all included data sources and their main characteristics. For each data source used, report reference information or contact name/institution, population represented, data collection method, year(s) of data collection, sex and age range, diagnostic criteria or measurement method, and sample size, as relevant.	An interactive, online data source tool that provides metadata for data sources by component, geography, cause, risk, or impairment has been developed	Online data citation tools: http://ghdx.healthdata.org/gbd-2016
6	Identify and describe any categories of input data that have potentially important biases (e.g., based on characteristics listed in item 5).	Summary of known biases by cause included in appendix	Appendix
For data inputs that contribute to the analysis but were not synthesised as part of the study:			
7	Describe and give sources for any other data inputs.	Included in online data source tool	http://ghdx.healthdata.org/gbd-2016
For all data inputs:			
8	Provide all data inputs in a file format from which data can be efficiently extracted (e.g., a spreadsheet as opposed to a PDF), including all relevant meta-data listed in item 5. For any data inputs that cannot be shared due to ethical or legal reasons, such as third-party ownership, provide a contact name or the name of the institution that retains the right to the data.	Downloads of input data available through online tools, including data visualisation tools and data query tools; input data not available in tools will be made available upon request	Online data visualisation tools, data query tools, and the Global Health Data Exchange
**Data analysis**			

9	Provide a conceptual overview of the data analysis method. A diagram may be helpful.	Flow diagrams of the overall methodological processes, as well as cause-specific modelling processes, have been provided	Main text (Methods) and appendix
10	Provide a detailed description of all steps of the analysis, including mathematical formulae. This description should cover, as relevant, data cleaning, data pre-processing, data adjustments and weighting of data sources, and mathematical or statistical model(s).	Flow diagrams and corresponding methodological write-ups for each cause, as well as the databases and modelling processes, have been provided	Main text (Methods) and appendix
11	Describe how candidate models were evaluated and how the final model(s) were selected.	Provided in the methodological write-ups	Appendix
12	Provide the results of an evaluation of model performance, if done, as well as the results of any relevant sensitivity analysis.	Provided in the methodological write-ups	Appendix
13	Describe methods for calculating uncertainty of the estimates. State which sources of uncertainty were, and were not, accounted for in the uncertainty analysis.	Appendix	Appendix
14	State how analytic or statistical source code used to generate estimates can be accessed.	Appendix	http://ghdx.healthdata.org/gbd-2016-code
**Results and Discussion**			
15	Provide published estimates in a file format from which data can be efficiently extracted.	GBD 2016 results are available through online data visualisation tools, the Global Health Data Exchange, and the online data query tool	Main text, and online data tools (data visualisation tools, data query tools, and the Global Health Data Exchange)
16	Report a quantitative measure of the uncertainty of the estimates (e.g. uncertainty intervals).	Uncertainty intervals are provided with all results	Main text, appendix, and online data tools (data visualisation tools, data query tools, and the Global Health Data Exchange)
17	Interpret results in light of existing evidence. If updating a previous set of estimates, describe the reasons for changes in estimates.	Discussion of methodological changes between GBD rounds provided in the narrative of the manuscript and appendix	Main text (Methods and Discussion) and appendix
18	Discuss limitations of the estimates. Include a discussion of any modelling assumptions or data limitations that affect interpretation of the estimates.	Discussion of limitations provided in the narrative of the main paper, as well as in the methodological write-ups in the appendix	Main text (Limitations) and appendix
